# ENOblock inhibits the pathology of diet-induced obesity

**DOI:** 10.1038/s41598-018-36715-3

**Published:** 2019-01-24

**Authors:** Haaglim Cho, Ji-Hyung Lee, JungIn Um, Sunwook Kim, Yukyung Kim, Woong-Hee Kim, Yong Sook Kim, Haushabhau S. Pagire, Jin Hee Ahn, Youngkeun Ahn, Young-Tae Chang, Da-Woon Jung, Darren R. Williams

**Affiliations:** 1 New Drug Targets Laboratory, School of Life Sciences, Gwangju Institute of Science and Technology, 1 Oryong-Dong, Buk-Gu, Gwangju 61005 Republic of Korea; 2 Cell Regeneration Research Center, Department of Cardiology, Cardiovascular Center, Chonnam National University Hospital, 42 Jebong-ro, Dong-gu, Gwangju 61469 Republic of Korea; 3 Department of Chemistry, Gwangju Institute of Science and Technology, 1 Oryong-Dong, Buk-Gu, Gwangju 61005 Republic of Korea; 4 Center for Self-assembly and Complexity, Institute for Basic Science (IBS), Pohang, 37673 Republic of Korea; 5 Department of Chemistry, Pohang University of Science and Technology (POSTECH), Pohang, 37673 Republic of Korea

## Abstract

Obesity is a medical condition that impacts on all levels of society and causes numerous comorbidities, such as diabetes, cardiovascular disease, and cancer. We assessed the suitability of targeting enolase, a glycolysis pathway enzyme with multiple, secondary functions in cells, to treat obesity. Treating adipocytes with ENOblock, a novel modulator of these secondary ‘moonlighting’ functions of enolase, suppressed the adipogenic program and induced mitochondrial uncoupling. Obese animals treated with ENOblock showed a reduction in body weight and increased core body temperature. Metabolic and inflammatory parameters were improved in the liver, adipose tissue and hippocampus. The mechanism of ENOblock was identified as transcriptional repression of master regulators of lipid homeostasis (*Srebp-1a* and *Srebp-1c*), gluconeogenesis (*Pck-1*) and inflammation (*Tnf-α* and *Il-6*). ENOblock treatment also reduced body weight gain, lowered cumulative food intake and increased fecal lipid content in mice fed a high fat diet. Our results support the further drug development of ENOblock as a therapeutic for obesity and suggest enolase as a new target for this disorder.

## Introduction

Enolase is an enzyme that catalyzes the conversion of 2-phosphoglycerate to phosphoenolpyruvate, the ninth and penultimate step of the anciently conserved glycolysis pathway^1^. It is ubiquitously expressed in human cells and proteomic meta-analysis identified α-enolase as the second protein to be differentially expressed in human pathologies, implicating enolase as an indicator of tissue dysfunction in multiple diseases^2^. Enolase has many diverse, secondary ‘moonlighting’^3^ functions that are unrelated to its catalytic activity, such as binding plasminogen (a key component of the fibrinolytic system) on the cell membrane, stabilizing the mitochondrial membrane, structural functions as a lens crystallin protein, and a repressor of gene expression in the nucleus^1,4,5^. Recently, we have developed the small molecule, ENOblock, as a chemical probe for elucidating the moonlighting functions of enolase in biological assays^4–7^. ENOblock binds enolase at the dimerization domain and induces nuclear localization, where it acts as a transcriptional repressor^6–8^. In contrast, other glycolytic enzymes that moonlight in the nucleus, such as phosphofructokinase, glyceraldehyde 3-phosphate dehydrogenase (GAPDH) and pyruvate kinase, activate gene expression^9^. ENOblock treatment increased enolase nuclear translocation in different cell types and was also effective *in vivo*, increasing enolase nuclear localization in mouse liver and kidney^7^. Previously, ourselves and others have used ENOblock to indicate non-glycolytic functions of enolase related to inflammation, cancer cell invasion/migration and glucose homeostasis^6,7,10–12^. Moreover, ENOblock treatment suppressed the expression of known enolase target genes, and produced anti-diabetic effects in a genetic model of diabetes^7^.

Obesity is a leading preventable cause of death and one of the most pressing health concerns of the 21st century^13^. It is classified as a disease^14^, with increasing rates in adults and children^15^, and numerous comorbidities, such as insulin resistance^16^, type 2 diabetes^17^, hypertension^18^, cardiovascular disease^19^, some types of cancer^19^, osteoarthritis^20^, asthma^21^, obstructive sleep apnea^22^ and psychological disorders^23^. The main treatment for obesity is lifestyle management (dieting and increased physical activity) although maintaining long-term weight loss is frequently difficult to achieve, with success ranging from 2–20%^24^. Bariatric surgery is the most effective treatment, but it is expensive and associated with complications^25^. Five medications are approved for the long-term treatment of obesity in the United States: orlistat, lorcaserin, liraglutide, phentermine–topiramate, and naltrexone–bupropion^26^. Unfortunately, these may produce significant side effects, such as gastrointestinal problems with orlistat^27^, and their long term effects on obesity-related comorbidities are not established^28^. Therefore, it is necessary to develop new therapeutics and drug targets for treating obesity.

In light of the relative lack of effective medicines and targets for treating obesity compared to diseases such as type 2 diabetes, we tested the pharmacological targeting of enolase moonlighting with ENOblock in a model of diet-induced obesity, wherein animals are fed a highly palatable, Western-style diet rich in fats and sugar. ENOblock was compared with rosiglitazone, a thiazolidinedione class of drug that can reduce the symptoms of prediabetes, but not obesity, and subsequently metformin, which is the most commonly prescribed anti-diabetic drug for obese patients that can also produce anti-obesity effects^29–31^. Our results show that ENOblock produced dramatic improvements on numerous pathological parameters of obesity by repressing the transcription of master regulators of adipogenesis, lipid homeostasis, inflammation and gluconeogenesis. These findings support the further development of ENOblock as a therapeutic for diet-induced obesity and implicate enolase as a novel target for treating this disorder.

## Results

### ENOblock treatment suppresses adipogenesis in differentiating white adipocyte and reduces mitochondrial membrane potential in white and brown adipocytes

To determine the potential of ENOblock as an anti-obesity therapeutic, the effect of this compound on adipogenesis-related gene expression was assessed by qPCR. The following genes were tested: adiponectin (*Adipoq*), adipocyte protein 2 (*Ap2*), peroxisome proliferator-activated receptor gamma (*Ppar-γ*), resistin (*Retn*), angiotensin (*Agt*), CCAAT/enhancer-binding protein-α (*Cebpa*) and CCAAT/enhancer-binding protein-β (*Cebpb*). Some classes of compounds that show anti-obesity effects in animal models, such the β-3 adrenergic agonist CL 316,243, upregulate oxidative phosphorylation or thermogenesis-related genes^32,33^. Therefore, genes regulating oxidative phosphorylation or thermogenesis were also assessed: (nuclear respiratory factor 1 (*Nrf1*), cytochrome c oxidase subunit VIIIb (*Cox8b*) and carnitine palmitoyltransferase I (*Cpt1b*)) or thermogenesis (uncoupling proteins 1–3 (*Ucp-1*, *Ucp-2*, *Ucp-3*), peroxisome proliferator-activated receptor gamma coactivator 1-alpha (*Pgc-1α*) and PR domain containing 16 (*Prdm16*)).

**Figure 1:**
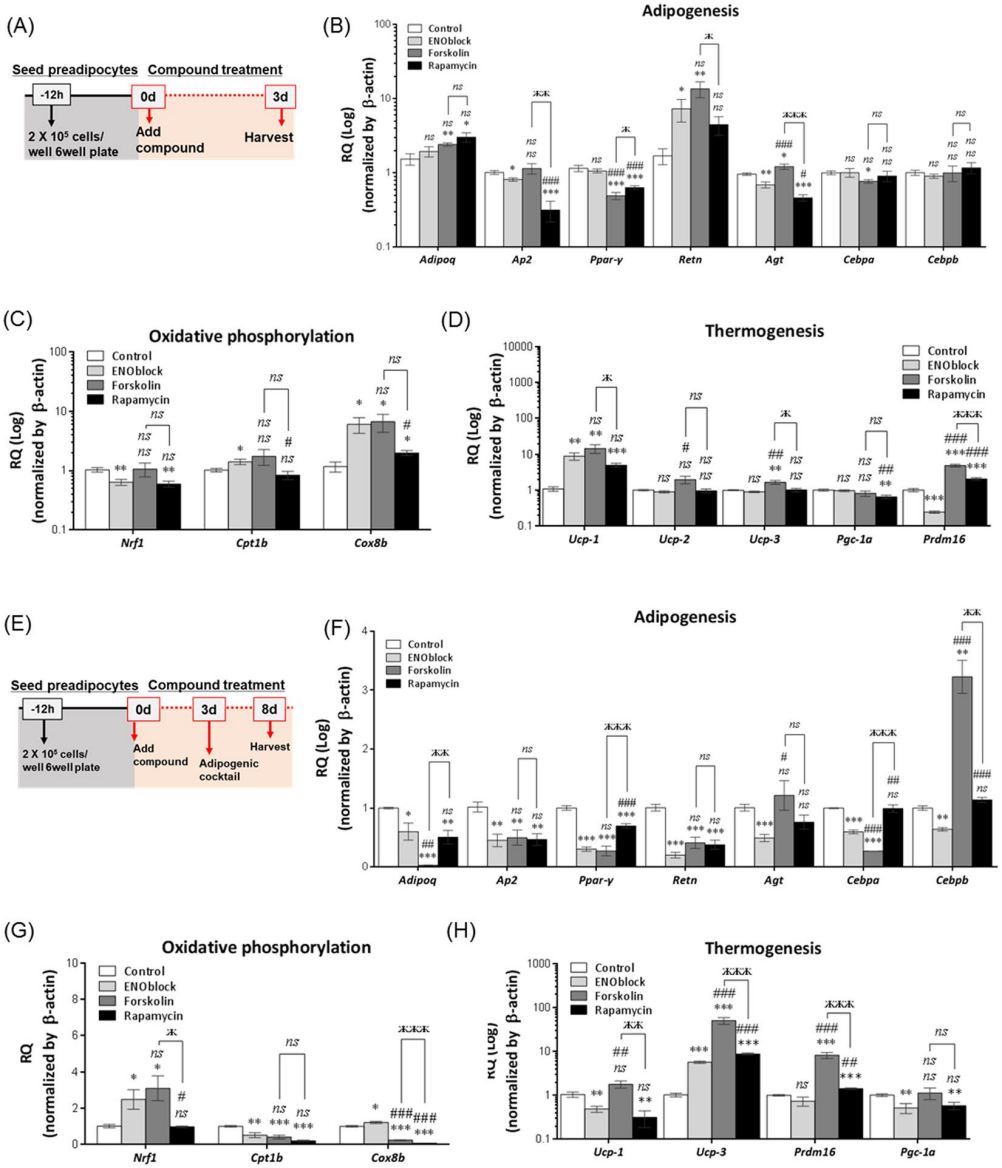
ENOblock effect on the induction of adipogenic gene expression in preadipocytes. (**A**) Schematic of the compound treatment protocol in primary WAT preadipocytes. (**B**) Effect of 72 h treatment with 10 µM forsoklin, 1 µM rapamycin or 10 µM ENOblock on the expression of adipogenesis regulatory genes. (**C**) Expression of oxidative phosphorylation regulatory genes after compound treatment. (**D**) Expression of thermogenesis regulatory genes after compound treatment. (**E**) Schematic of the compound pre-treatment protocol in WAT preadipocytes undergoing adipogenic differentiation. (**F**) Effect of treatment with 10 µM forsoklin, 1 µM rapamycin or 10 µM ENOblock on the expression of adipogenesis regulatory genes in differentiating preadipocytes. (**G**) Expression of oxidative phosphorylation regulatory genes after compound treatment. (**H**) Expression of thermogenesis regulatory genes after compound treatment. The treatment concentrations of forskolin, rapamycin or ENOblock were based on the following references^7,87,88^. n = 9; ns: not significantly different. *, ** or ***: significantly different from the corresponding ‘Control’ or ‘Untreated’ respectively with p < 0.05, p < 0.01 or p < 0.001; ## or ###: significantly different from the corresponding ‘ENOblock’, ж, жж or жжж: significantly different from the corresponding ‘Forskolin’.

As a first test, murine primary cultures of white preadipocytes were treated with ENOblock for 72 h (Fig. 1A). The effects on gene expression pattern was compared with preadipocytes treated with known compounds that enhance thermogenesis (forskolin^34–36^) or block adipogenesis (rapamycin^37,38^). After 72 h treatment, ENOblock treated preadipocytes showed no significant change in the expression of the adipogenesis regulatory genes, *Adipoq*, *Ppar-γ*, *Cebpa* and *Cebpb*, down-regulated expression of *Ap2* and *Agt*, and upregulated expression of *Retn* (Fig. 1B). ENOblock treatment upregulated expression of the markers of oxidative phosphorylation, *Cox8b* and *Cpt1b*, and down-regulated *Nrf1*. The thermogenesis marker, *Ucp-1* was upregulated after ENOblock treatment, whereas *Prdm16* was down-regulated and *Ucp-2*, *Ucp-3* and *Pgc-1α* showed no significant change in expression (Fig. 1C,D).

To assess the effect of ENOblock on the induction of adipogenesis, the preadipocytes were treated with ENOblock for 72 h, followed by adipogenic factors for 5 days (Fig. 1E–H). ENOblock treated cells showed significant downregulation of the adipogenesis genes *Adipoq*, *Ap2*, *Ppar-γ*, *Retn*, *Agt*, *Cebpa* and *Cebpb*. Treatment with rapamycin produced downregulation of *Adipoq*, *Ap2*, *Ppar-γ* and *Retn*, but not *Agt*, *Cebpa* and *Cebpb*. ENOblock treatment upregulated the oxidative phosphorylation marker genes *Nrf1* and Cox8b, and downregulated *Cpt1b*. ENOblock treatment increased expression of the thermogenesis marker, *Ucp-3*, but not *Ucp-1, Prdm16* or *Pgc-1α*. Forskolin treatment increased expression of the markers *Ucp-3* and *Prdm16* (*Ucp-2* expression was not detectable in the differentiating adipocytes using qPCR).

**Figure 2:**
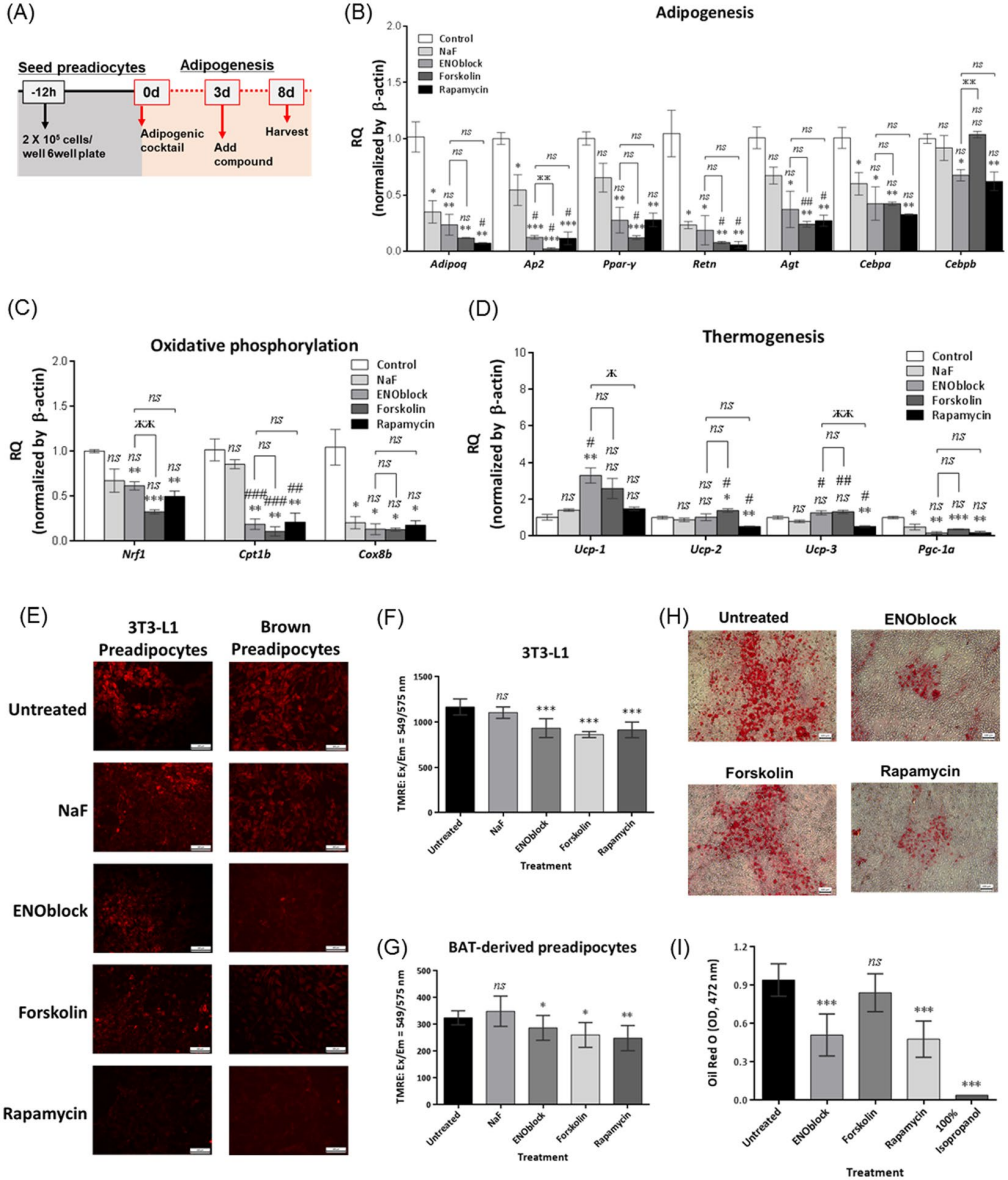
Influence of ENOblock on the adipogenic program in differentiating preadipocytes and mitochondrial membrane potential. (**A**) Schematic of the compound treatment protocol in primary cultures of differentiating white adipocytes. (**B**) Effect of 72 h treatment with 10 µM forsoklin, 1 µM rapamycin,10 µM ENOblock or 1 mM NaF on the expression of adipogenesis regulatory genes in differentiating adipocytes. (**C**) Expression of oxidative phosphorylation regulatory genes. (**D**) Expression of thermogenesis regulatory genes. (**E**) Live cell imaging of TMRE fluorescence to visualize mitochondrial membrane potential in 3T3-L1 white preadipocytes and brown preadipocytes after treatment with 10 µM ENOblock, 1 mM NaF, 10 µM forsoklin or 1 µM rapamycin for 72 h. (**F**) Quantification of mitochondrial membrane potential in 3T3-L1 preadipocytes. (**G**) Quantification of mitochondrial membrane potential in brown preadipocytes. (**H**) Oil red O staining of 3T3-L1 white preadipocytes treated with 10 µM ENOblock, 1 mM NaF, 10 µM forsoklin or 1 µM rapamycin for 72 h and adipogenic factors for 5 days. (**I**) Quantification of oil red O staining in the treated adipocytes. n = 9; ns: not significantly different. *, ** or ***: significantly different from the corresponding ‘Control’ or ‘Untreated’ respectively with p < 0.05, p < 0.01 or p < 0.001; ## or ###: significantly different from the corresponding ‘NaF’ sample with *p* < 0.01 or *p* < 0.001; ж, жж or жжж: significantly different from the corresponding ‘ENOblock’ sample respectively with *p* < 0.05, *p* < 0.01 or *p* < 0.001.

To investigate the effect of ENOblock on adipocytes in the process of adipogenesis, primary white adipocytes were treated with adipogenic factors for 72 h, followed by ENOblock treatment for 5 days (Fig. 2A–D). For this test, the effect of ENOblock treatment was compared with NaF, an enolase enzyme inhibitor that, unlike ENOblock, does not induce enolase nuclear translocation^7^. ENOblock treatment inhibited expression of the adipogenic genes *Adipoq*, *Ap2*, *Ppar-γ*, *Retn*, *Agt*, *Cebpa* and *Cebpb*. Treatment with NaF downregulated *Adipoq*, *Ap2*, *Retn* and *Cebpa*, but not *Ppar-γ* and *Cebpb*. Similar to ENOblock, rapamycin treatment also downregulated expression of all 7 adipogenesis-related genes. ENOblock down-regulated expression the oxidative phosphorylation markers *Nrf1, Cox8b* and *Cpt1b*, and upregulated expression of the thermogenesis marker, *Ucp-1*, but not *Ucp-*2, *Ucp-3* and *Prdm16*. Forskolin treatment also upregulated *Ucp-1* and down-regulated *Nrf1, Cox8b* and *Cpt1b* (Fig. 2C,D). NaF treatment down-regulated *Cox8b* and did not significantly influence expression of *Nrf1, Cpt1b* or *Ucp-1*. Overall, these results indicate that ENOblock is effective at blocking adipogenesis-related gene expression in white adipocytes undergoing differentiation. In differentiating adipocytes and preadipocytes, ENOblock treatment upregulated expression of the thermogenesis genes, *Ucp-1*, although there was no concomitant upregulation of genes regulating oxidative phosphorylation.

The effects of ENOblock treatment on adipogenesis, oxidative phosphorylation and thermogenesis was also tested in primary cultures of differentiating brown preadipocytes derived from brown adipose tissue (BAT) (Supplementary Fig. 3). The adipogenesis genes *Adipoq*, *Ap2*, *Ppar-γ*, *Retn*, *Agt* and *Cebpa* were not significantly affected by ENOblock treatment. Oxidative phosphorylation markers *Nrf1* and *Cpt1b* were down-regulated by ENOblock and expression of the thermogenesis markers *Ucp-1*, *Ucp-*2 and *Ucp-3* were not significantly affected (Supplementary Fig. 3A–D). This result indicates that ENOblock is more effective at blocking adipogenesis gene-related expression in white adipocytes compared to brown adipocytes.

Anti-obesity agents can induce thermogenesis in brown adipose tissue (BAT) and ‘browning’ of white adipose tissue (WAT), which is detected as proton leak in the inner mitochondrial membrane^33,39,40^. 3T3-L1 white preadipocytes were treated with ENOblock, NaF, rapamycin, or forskolin. Mitochondrial membrane potential (an indicator of proton leak in the inner mitochondrial membrane) was measured using tetramethylrhodamine, ethyl ester (TMRE, an indicator of mitochondrial membrane potential^41^). 3T3-L1 and brown preadipocytes treated with ENOblock for 72 h showed decreased membrane potential (Fig. 2E,F). The inhibitory effect of ENOblock on membrane potential was also confirmed in white primary preadipocytes using automated microscopy (Supplementary Fig. 3E,F). Treatment with forskolin or rapamycin also reduced membrane potential in the preadipocytes. NaF treatment did not reduce membrane potential. Based on this result, these compounds were further tested in primary cultures of BAT derived preadipocytes. In brown preadipocytes, ENOblock, rapamycin and forskolin, significantly reduced mitochondrial membrane potential (Fig. 2E–G). To confirm the ENOblock-mediated adipogenesis gene suppression inhibits adipogenesis, differentiating cultures of 3T3-L1 white preadipocytes were treated with ENOblock, forskolin or rapamycin for 72 hours and adipogenic factors for 5 days, and stained with Oil Red O to visualize lipid accumulation. Treatment with ENOblock or forskolin reduced lipid accumulation in the differentiating adipocytes (Fig. 2H,I).

In human hepatocytes treated with ENOblock, enolase was observed to accumulate in the nucleus (Supplementary Fig. 1A). This effect was not observed after rosiglitazone treatment. Although the exact mechanism of enolase nuclear translocation is unknown^42^, the *O*-GlcNAc modification has been reported on enolase and is linked to nuclear localization^43,44^. Treatment with OSMI-1 was shown to reduce enolase localization in the presence of ENOblock (Supplementary Fig. 1B). Inhibiting enolase nuclear import with OSMI-1 was also shown to reduce the effects of ENOblock on SREBP-1a, -1c and SREBP-2 expression (inserted as Supplementary Fig. 1D). Additionally, we observed that OSMI-1 treatment reduced the inhibitory effect of ENOblock on lipid accumulation in differentiating adipocytes (inserted as Supplementary Fig. 1C). OSMI-1 treatment also inhibited the negative effect of ENOblock on SPREBP expression (Supplementary Fig. 1D). Moreover, siRNA mediated silencing of enolase also inhibited the negative effect of ENOblock on SREBP expression (Supplementary Fig. 2A,B). Chromatin immunoprecipitation assay of the SREBP-1 and -2 upstream promoters indicated enolase direct binding, with a higher detection signal for the SREBP-2 promoter compared to SREBP-1 (Supplementary Fig. 2C).

### ENOblock treatment reduces weight gain, recovers body temperature and prevents hyperglycemia in diet-induced obese mice

**Figure 3:**
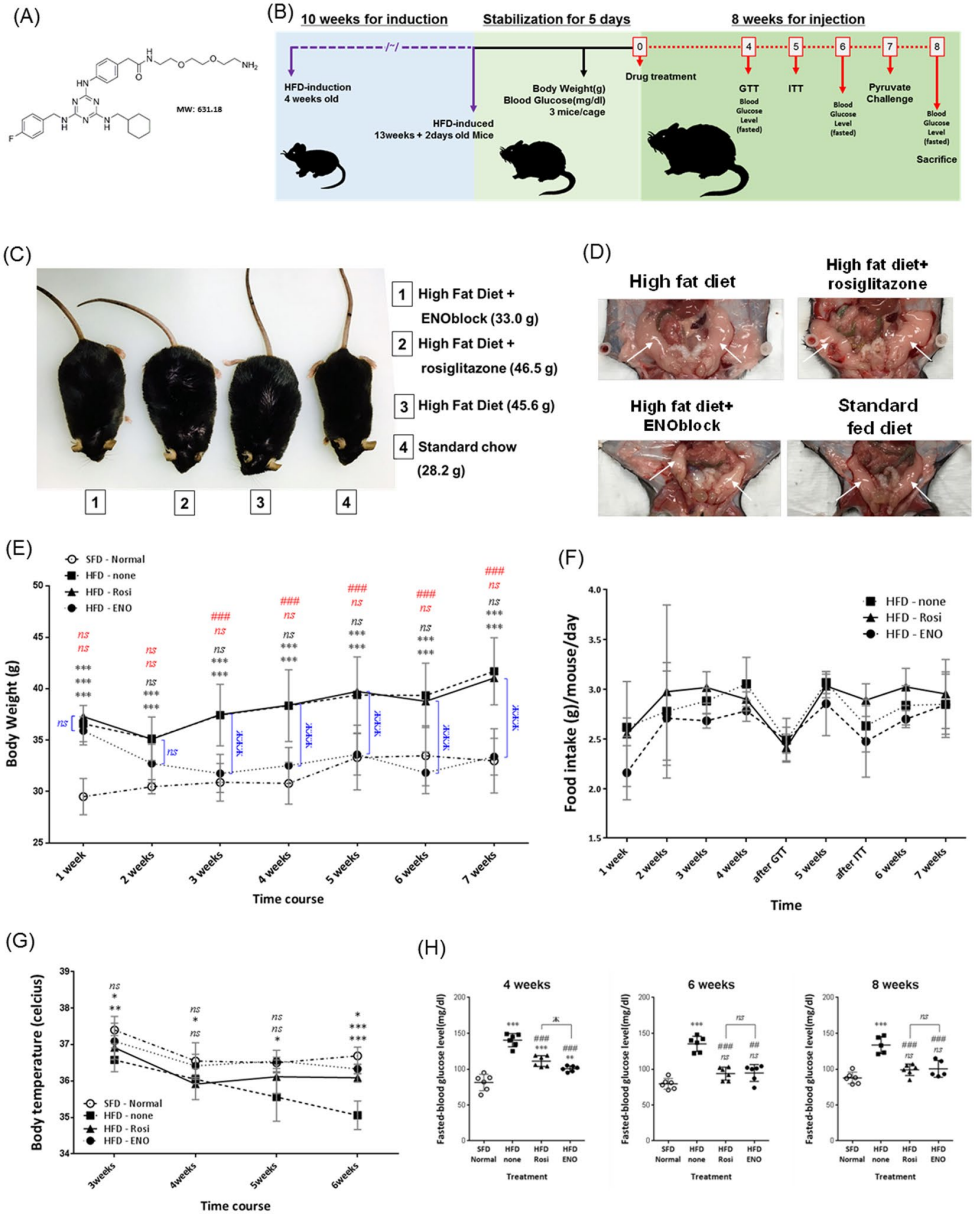
Effect of ENOblock treatment on visceral fat, weight, body temperature and fasted glucose level in obese mice. (**A**) Chemical structure of ENOblock. (**B**) Schematic of the ENOblock treatment protocol in HFD mice. (**C**) Photograph showing the overall effect of 8 weeks treatment with ENOblock or rosiglitazone in HFD mice. (**D**) Photograph of the dissected abdomen showing visceral fat tissues in the treated mice (indicated with white arrows). (**E**) Effect of ENOblock or rosiglitazone treatment on body weight in HFD mice. n = 6. (**F**) Food intake in the treated mice. n = 6. (**G**) Body temperature of the mice during drug treatment. n = 6. (**H**) Fasted blood glucose level in the sera of HFD mice after 4, 6, and 8 weeks treatment with ENOblock or rosiglitazone. SFD = mice fed standard chow; HFD = high fat diet-fed mice; HFD-ENO = ENOblock treated HFD mice; HFD-Rosi = rosiglitazone treated HFD mice. n = 6; ns: not significantly different. *, ** or ***: significantly different from the corresponding ‘SFD-Normal’ or ‘SFD-Control’ (Standard Fat Diet-none-treated normal healthy mouse group) respectively with *p* < 0.05, *p* < 0.01 or *p* < 0.001; ## or ###: significantly different from the corresponding ‘HFD-none’ or ‘HFD-Control’ (HFD-non-treated control mouse group) sample with *p* < 0.01 or *p* < 0.001; ж, жж or жжж: significantly different from the corresponding ‘HFD-Rosi’ sample respectively with *p* < 0.05, *p* < 0.01 or *p* < 0.001. The copyright holder (Mrs Hyunju Park) has granted permission to Springer Nature Limited to publish the images of the mice in Fig. 3 of the manuscript entitled “ENOblock inhibits the pathology of diet-induced obesity” by Cho, *et al*., under a CC BY open access license.

The chemical structure of ENOblock is shown in Fig. 3A. A schematic of the treatment protocol to investigate the effects of ENOblock in the high fat diet (HFD) induced model of obesity is shown in Fig. 3B. At the end of the 8 weeks’ drug treatment regime, ENOblock treated mice showed reduced body weight compared to their untreated or rosiglitazone-treated counterparts (Fig. 3C–E). During the 8 weeks of drug treatment, weight gain in the ENOblock treated HFD mice was reduced compared to untreated and rosiglitazone-treated HFD mice (Fig. 3D,E). The reduction in body weight between ENOblock treated and untreated HFD mice achieved statistical difference after three weeks. After seven weeks of treatment, ENOblock HFD mouse body weight was not significantly different to mice fed a standard chow diet (SFD group) (Fig. 3E). Measurement of food intake showed no significant difference between the treatment groups (Fig. 3F). ENOblock treatment produced significantly elevated body temperature compared to untreated and rosiglitazone-treated mice, which became apparent at 6 weeks of drug treatment (Fig. 3G). The body temperature in ENOblock treated mice was not significant compared to the SFD mice at 6 weeks of drug treatment, whereas body temperature in the rosiglitazone-treated mice was significantly lower than SFD mice (Fig. 3G). ENOblock and rosiglitazone-treated mice showed significantly reduced fasted blood glucose level compared to HFD mice at 3, 5 and 7 weeks of drug treatment (Fig. 3H).

### ENOblock treatment improved glucose-, insulin-, and pyruvate tolerance, and reduced hyperinsulinemia in obese mice

**Figure 4:**
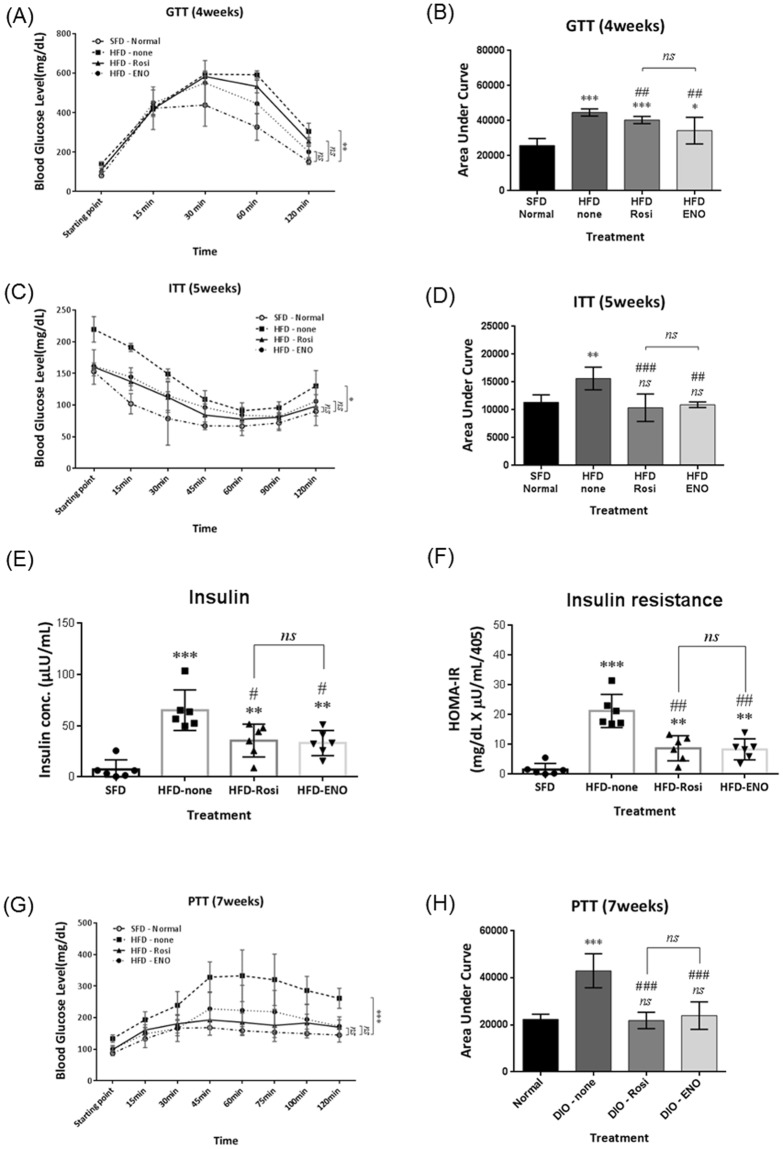
Effect of ENOblock treatment on glucose homeostasis, insulin resistance and gluconeogenesis in diet-induced obese mice. (**A**,**B**) Glucose tolerance test (GTT) and area under the curve (AUC) for HFD mice treated with ENOblock or rosiglitazone for 4 weeks. (**C**,**D**) Insulin tolerance test (ITT) and AUC for the treated HFD mice after 5 weeks of ENOblock or rosiglitazone treatment. (**E**,**F**) Insulin serum level and determination of insulin resistance level in HFD mice after 8 weeks of ENOblock or rosiglitazone treatment. (**G**,**H**) Pyruvate tolerance test (PTT) after 7 weeks of drug treatment to determine gluconeogenesis level. SFD = mice fed standard chow; HFD = high fat diet-fed mice; HFD-ENO = ENOblock treated HFD mice; HFD-Rosi = rosiglitazone treated HFD mice. n = 6; ns: not significantly different. *, ** or ***: significantly different from the corresponding ‘SFD-Normal’ or ‘SFD-Control’ (Standard Fat Diet-none-treated normal healthy mouse group) respectively with *p* < 0.05, *p* < 0.01 or *p* < 0.001; ## or ###: significantly different from the corresponding ‘HFD-none’ or ‘HFD-Control’ (HFD-non-treated control mouse group) sample with *p* < 0.01 or *p* < 0.001; ж, жж or жжж: significantly different from the corresponding ‘HFD-Rosi’ sample respectively with *p* < 0.05, *p* < 0.01 or *p* < 0.001.

Mice were subjected to a glucose tolerance test (GTT) at 4 weeks of treatment. ENOblock- and rosiglitazone-treated mice showed improved glucose tolerance compared to untreated HFD mice (Fig. 4A,B). An insulin tolerance test (ITT) was carried out after 5 weeks of drug treatment. Compared to HFD mice, ENOblock- and rosiglitazone-treated mice showed improved insulin tolerance, which was not significantly different to insulin tolerance in SFD mice (Fig. 4C,D). Hyperinsulinemia was also reduced in the ENOblock- and rosiglitazone-treated mice compared to HFD mice, along with a concomitant reduction in homeostatic model assessment – insulin resistance (HOMA-IR) (Fig. 4E,F). The pyruvate tolerance test (PTT) was administered after 7 weeks of drug treatment. ENOblock- and rosiglitazone-treated HFD mice showed an improved blood glucose response after pyruvate challenge compared to the untreated HFD mice (Fig. 4G–H). Blood glucose level after PTT showed no statistical significance between SFD mice and the ENOblock-treated or rosiglitazone-treated HFD mice.

### ENOblock treatment prevents steatosis and fibrosis in the liver of obese mice

**Figure 5:**
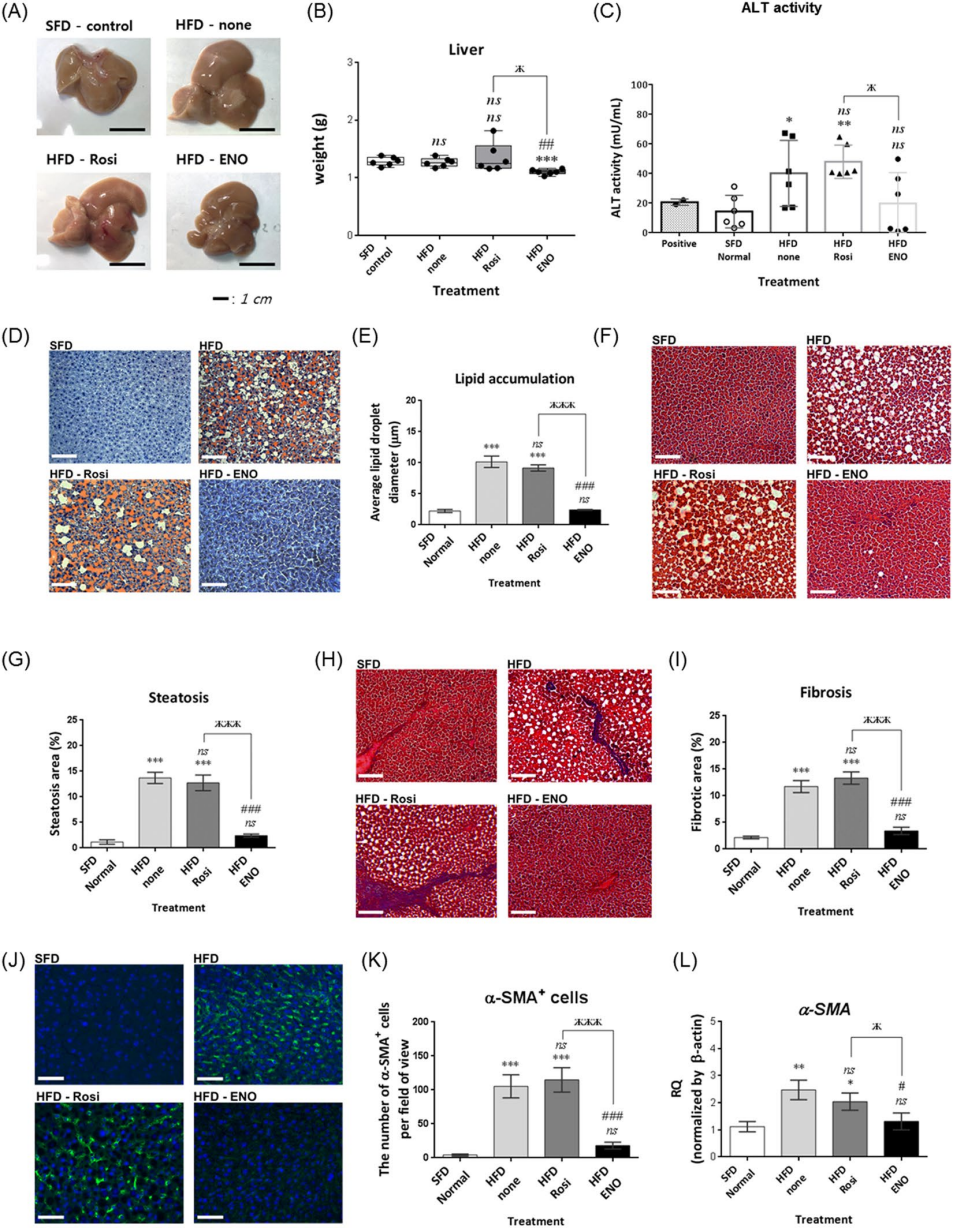
Consequence of ENOblock treatment on liver pathology in obese mice. (**A**) Representative photographs of the liver in HFD mice treated with ENOblock or rosiglitazone. Age-match SFD liver is included for comparison. (**B**) Liver weight in the treated mice. n = 6. (**C**) Serum levels of the liver enzyme alanine aminotransferase (ALT). n = 6. (**D**) Oil red O staining to visualize lipid accumulation in liver tissue. (**E**) Quantification of lipid accumulation. n = 30 randomly selected lipid droplets in 5 randomly captured microscope images from 5 mouse livers/treatment group. (**F**) H&E staining to visualize microsteatosis. (**G**) Quantification of microsteatosis. n = 10 different regions of 5 mouse livers per treatment group. (**H**) Masson’s Trichrome staining to indicate fibrosis (blue color) in the liver tissue. (**I**) Quantification of fibrosis. n = 8 to 10 randomly captured microscope images from sections, prepared from 5 mouse livers per treatment group. (**J**) α-SMA immunostaining to detect hepatic stellate cells. (**K**) Quantification of hepatic stellate cells. n = 10 randomly captured microscope images of cryo-sections from 5 mouse livers per treatment group. (**L**) qPCR analysis of *α-Sma* expression. n = 5 For (**D**), (**F**), (**H**) and (**J**); Scale bar = 100 µm. ns: not significantly different. *, ** or ***: significantly different from the corresponding ‘SFD-Normal’ or ‘SFD-Control’ (Standard Fat Diet-none-treated normal healthy mouse group) respectively with *p* < 0.05, *p* < 0.01 or *p* < 0.001; ## or ###: significantly different from the corresponding ‘HFD-none’ or ‘HFD-Control’ (HFD-non-treated control mouse group) sample with *p* < 0.01 or *p* < 0.001; ж, жж or жжж: significantly different from the corresponding ‘HFD-Rosi’ sample respectively with *p* < 0.05, *p* < 0.01 or *p* < 0.001.

Photographs of representative, dissected liver tissue from the HFD mice after 8 weeks of ENOblock treatment are shown in Fig. 5A. HFD and rosiglitazone treated mice showed visibly paler patches on the liver tissue compared to SFD and ENOblock-treated HFD mice. ENOblock-treated HFD mice had significantly smaller liver weight compared to the HFD and SFD mice (Fig. 5B). A serum alanine aminotransferase (ALT) assay was carried out to assess potential hepatotoxicity caused by ENOblock treatment. Untreated HFD and rosiglitazone-treated HFD mice showed significantly elevated serum ALT compared to SFD mice at 8 weeks of drug treatment. ALT level in the ENOblock-treated mice was not significantly elevated compared to the SFD mice (Fig. 5C).

Oil red O staining was used to assess liver lipid accumulation. HFD mice showed significant lipid accumulation compared to SFD mice, which was inhibited by ENOblock treatment (Fig. 5D,E). Rosiglitazone treatment did not reduce lipid accumulation. H&E staining indicated that HFD mice developed liver microsteatosis (Fig. 5F,G). ENOblock treatment reduced microsteatosis, whereas rosiglitazone treatment had no significant effect (Fig. 5F,G). Masson’s Trichrome staining showed the significant development of liver fibrosis in HFD mice. ENOblock treatment, but not rosiglitazone, reduced fibrosis to the level observed in SFD mice (Fig. 5H,I). The development of liver fibrosis by diet-induced obesity is associated with the activation of hepatic stellate cells, which can be detected by immunostaining for alpha smooth muscle actin (α-SMA)^45^. HFD mice showed increased levels of hepatic stellate cells compared to SFD mice. ENOblock, but not rosiglitazone, reduced stellate cell numbers to the level observed in the SFD mice (Fig. 5J,K). The ability of ENOblock to reduce the development of fibrosis in the liver of HFD mice was confirmed by qPCR and western blot analysis of α-SMA expression (Fig. 5L and Supplementary Fig. 4).

### ENOblock treatment prevents inflammation and induces suppressors of lipid homeostasis and gluconeogenesis in the liver of obese mice

**Figure 6:**
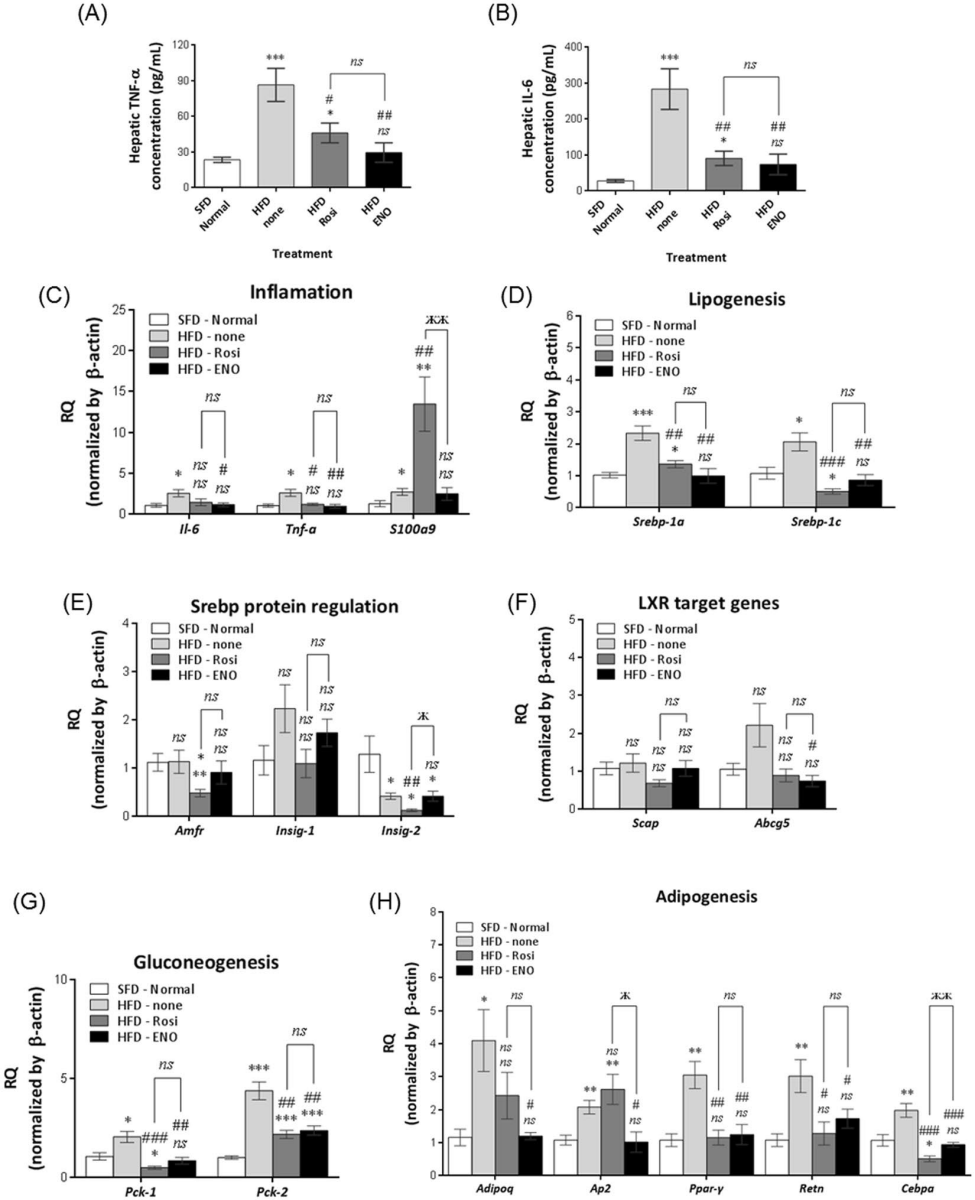
Effect of ENOblock on indicators of liver inflammation, lipogenesis and gluconeogenesis. (**A**,**B**) ELISA analysis of the inflammatory markers TNF-α and IL-6 in the liver of SFD, HFD and HFD mice treated with ENOblock or rosiglitazone for 8 weeks. n = 5 (**C**) Expression of the inflammatory markers *Il-6*, *Tnf-α* and *s100a9* in liver tissue of the treated mice. (**D**) qPCR analysis of the expression of the lipid homeostasis regulators, *Srebp-1a* and *Srebp-1c*. (**E**) qPCR analysis of the expression of the regulators of *Srebp-1a* and *Srebp-1c* processing, *Amfr, Insig-1* and *Insig-2*. (**F**) Expression of the LXR target genes, *Scap* and Abcg5. (**G**) Expression of the gluconeogenesis regulators, *Pck-1* and *Pck-2*. (**F**) Expression of the adipogenesis markers *Adipoq*, *Ap2*, *Ppar-γ*, *Retn* and *Cebpa*. SFD = mice fed standard chow; HFD = high fat diet-fed mice; HFD-ENO = ENOblock treated HFD mice; HFD-Rosi = rosiglitazone treated HFD mice. For (**C**–**F**) n = 5. ns: not significantly different. *, ** or ***: significantly different from the corresponding ‘SFD-Normal’ or ‘SFD-Control’ (Standard Fat Diet-none-treated normal healthy mouse group) respectively with *p* < 0.05, *p* < 0.01 or *p* < 0.001; ## or ###: significantly different from the corresponding ‘HFD-none’ or ‘HFD-Control’ (HFD-non-treated control mouse group) sample with *p* < 0.01 or *p* < 0.001; ж, жж or жжж: significantly different from the corresponding ‘HFD-Rosi’ sample respectively with *p* < 0.05, *p* < 0.01 or *p* < 0.001.

HFD-induced liver steatosis can progress to chronic inflammation and cirrhosis^45^. Assessment of liver inflammatory markers indicated that HFD increased expression of interleukin-6 (*Il-6*) and tumor necrosis factor-alpha (*Tnf-α*). ENOblock or rosiglitazone treatment reduced the expression of *Tnf-α* and *Il-6*, which were normalized compared to SFD mice (Fig. 6A,B). S100 calcium-binding protein A9 (*S100a9*) regulates myeloid cell function and is a biomarker for non-alcoholic steatohepatitis^46^. *S100a9* expression was increased in HFD compared to SFD mice. Rosiglitazone treatment further increased *S100a9* expression in the HFD mice, whereas ENOblock treatment had no effect (Fig. 6C).

Sterol regulatory element-binding proteins (*Srebp-1a* and *Srebp-1c*) are key regulators of lipid synthesis^47^. *Srebp-1a* and *Srebp-1c* expression was upregulated in the liver of HFD mice compared to SFD mice. ENOblock treatment inhibited *Srebp-1a* and *Srebp-1c* expression (Fig. 6D). Rosiglitazone treatment also inhibited *Srebp-1a* and *Srebp-1c* expression. *Insig-1* and *Insig-2* proteins block the maturation of *Srebp-1a* and *Srebp-1c* in the Golgi^47^, and are in turn regulated by autocrine motility factor receptor, isoform 2 (*Amfr*, also known as *Gp-78*)^48^. ENOblock treatment did not significantly affect the expression of *Amfr* and *Insig-1*, but did inhibit *Insig-2* expression (Fig. 6E). In addition, expression of the liver X receptor (LXR) target genes, *Scap* and *Abcg5*, were either unaffected or inhibited by ENOblock treatment, respectively (Fig. 6F). The onset of prediabetes in obesity is associated with dysregulated gluconeogenesis, which is positively regulated by phosphoenolpyruvate carboxykinase (*Pck*)^49^. HFD mice showed elevated expression of *Pck-1* and *Pck-2* compared to SFD mice. ENOblock or rosiglitazone treatment reduced *Pck-1* and *Pck-2* expression in the HFD mice (Fig. 6G).

The mechanism by which ENOblock inhibits steatosis in HFD mice was assessed by measuring the expression of key regulators of adipogenesis: *Adipoq*, *Ap2*, *Ppar-γ, Retn* and *Cebpa*^33^. HFD mice showed upregulated expression of all 5 adipogenic genes compared to SFD mice. ENOblock treatment reduced the expression of all 5 genes to the level observed in SFD mice (Fig. 6H). Rosiglitazone treatment reduced the expression of *Ppar-γ*, *Retn* and *Cebpa* in HFD mice, but did not significantly reduce the expression of *Adipoq* and *Ap2*.

### ENOblock prevents mitochondrial loss and reduces inflammatory marker expression in the hippocampus of obese mice

**Figure 7:**
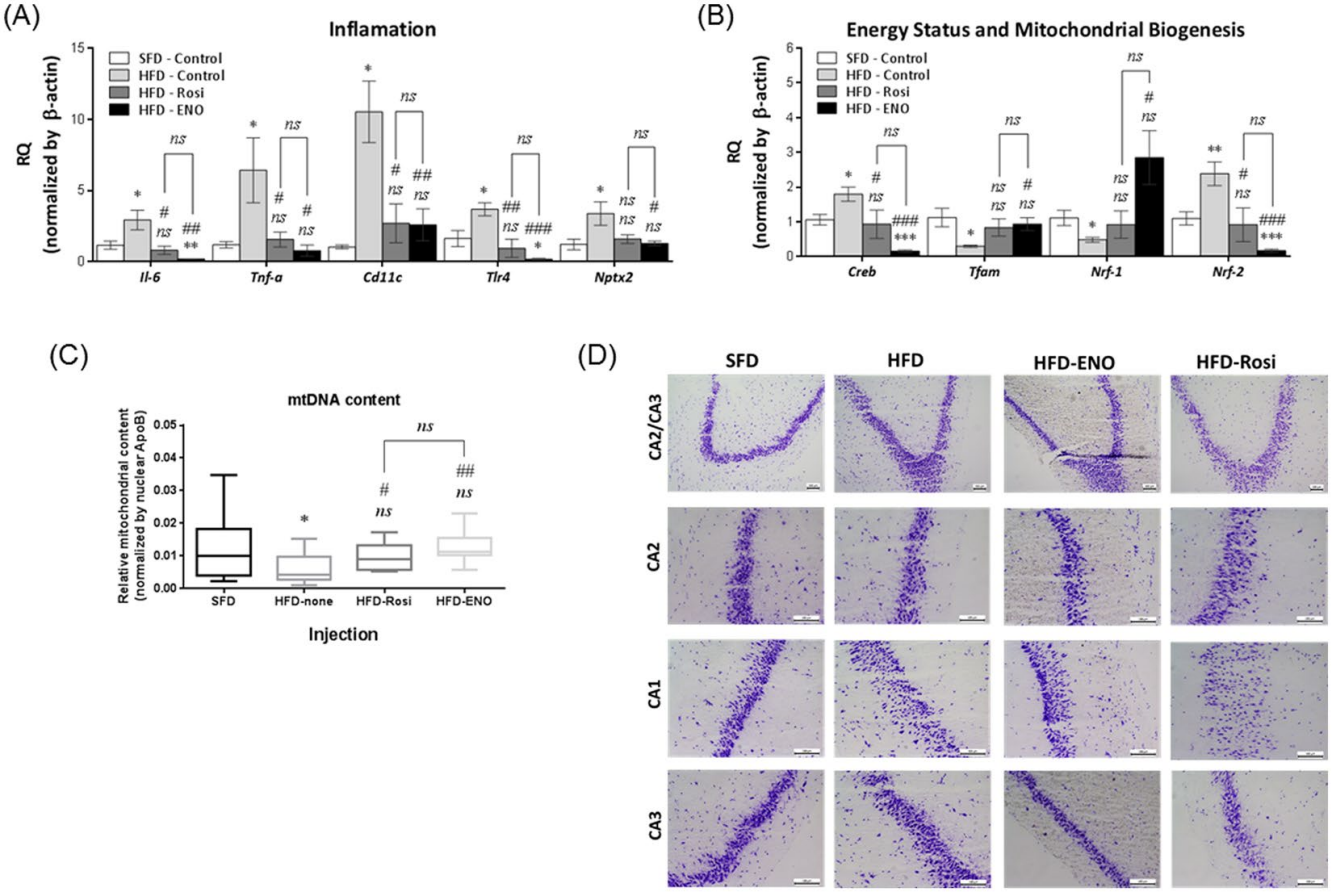
Hippocampus expression of inflammatory markers and sensors of energy status, mitochondrial content and neuronal histology in obese mice after ENOblock treatment. (**A**) Expression of the inflammatory markers *Il-6*, *Tnf-*α, *Cd11c*, *Tlr-4* and *Nptx2* in the hippocampus. (**B**) Expression of the energy status sensor, *Creb*, and the regulators of mitochondrial biogenesis, *Tfam*, *Pgc1α, Pgc1β, Nrf1*, and *Nrf2*. (**C**) Mitochondrial DNA content in the hippocampus. (**D**) Nissl staining of neurons in the CA1, CA2 and CA3 regions of the hippocampus. Note the disorganized neuronal structures in the CA1 region of rosiglitazone treated mice. Scale bar = 100 µm. SFD = mice fed standard chow; HFD = high fat diet-fed mice; HFD-ENO = ENOblock treated HFD mice; HFD-Rosi = rosiglitazone treated HFD mice. For (**A**–**D**) n = 5 ns: not significantly different. *, ** or ***: significantly different from the corresponding ‘SFD-Normal’ or ‘SFD-Control’ (Standard Fat Diet-none-treated normal healthy mouse group) respectively with *p* < 0.05, *p* < 0.01 or *p* < 0.001; ## or ###: significantly different from the corresponding ‘HFD-none’ or ‘HFD-Control’ (HFD-non-treated control mouse group) sample with *p* < 0.01 or *p* < 0.001; ж, жж or жжж: significantly different from the corresponding ‘HFD-Rosi’ sample respectively with *p* < 0.05, *p* < 0.01 or *p* < 0.001.

Obesity has been linked with hippocampal dysfunction and memory impairment, which is thought to result from HFD-induced inflammatory responses in the hippocampus^50,51^. After 8 weeks, HFD mice showed increased expression of the hippocampal inflammatory markers toll-like receptor (*Tlr4*), *Il-6*, *Tnf-α* and CD11c compared to SFD mice. ENOblock treatment reduced the expression of these inflammatory markers in HFD mice to the level observed in SFD mice (for *Tnf-α* and *Cd11c*) or lower than SFD mice (for *Tlr4* and *Il-6*) (Fig. 7A). Rosiglitazone treatment also reduced hippocampal inflammatory marker expression. Neuronal pentraxin-2 (*Nptx2*) regulates synaptic plasticity and is a pro-inflammatory biomarker of non-apoptotic neuronal cell death^52^. *Nptx2* expression was upregulated in the HFD mice compared to SFD mice. ENOblock treatment reduced *Nptx2* expression in the HFD mice to the level observed in SFD mice (Fig. 7A). Rosiglitazone also reduced *Nptx2* expression in the HFD mice.

HFD is known to reduce mitochondrial mass in various cell types, which can produce changes in brain energetics and infrastructure^53,54^. Transcription factor A of the mitochondria (*Tfam*) is positively linked to the regulation of mitochondrial genome copy number^55^. *Tfam* expression was reduced in the hippocampus of HFD mice compared to SFD mice. ENOblock treatment increased *Tfam* expression to the levels observed in SFD mice (Fig. 7B). Rosiglitazone treatment also increased *Tfam* expression in HFD mice. The transcription factor cAMP response element-binding protein (*Creb*) is sensitive to alterations in the energy status of neuronal cells^56^. *Creb* expression was increased in the HFD mice compared to SFD mice. ENOblock, but not rosiglitazone, treatment reduced *Creb* expression in HFD mice (Fig. 7B). Transcription factor nuclear respiratory factors (*Nrf-1* and *Nrf-2*) regulates neurite outgrowth and mitochondrial biogenesis^57,58^. *Nrf-1* expression was reduced in HFD mice and ENOblock treatment significantly increased *Nrf-1* expression (Fig. 7B). In contrast, *Nrf-2* expression was elevated in HFD mice and reduced by ENOblock treatment (Fig. 7B). Rosiglitazone treatment did not affect *Nrf-1* expression but, similar to ENOblock treatment, reduced the expression of *Nrf-2*. The negative effects of HFD on hippocampal bioenergetics was indicated by a reduction in mtDNA content. ENOblock treatment prevented the reduction of mtDNA content (Fig. 7C). Rosiglitazone treatment also improved mtDNA content compared to HFD mice, but with less significance than ENOblock treatment (*p* < 0.05 compared with *p* < 0.01, respectively). Nissl staining of hippocampal neurons did not show histological differences in neural survival in SFD, HFD or ENOblock-treated HFD mice (Fig. 7D). Rosiglitazone-treated mice showed disrupted Nissl staining in the CA1 region of the hippocampus.

### ENOblock lowers serum lipids and adiposity in diet-induced obese mice

**Figure 8:**
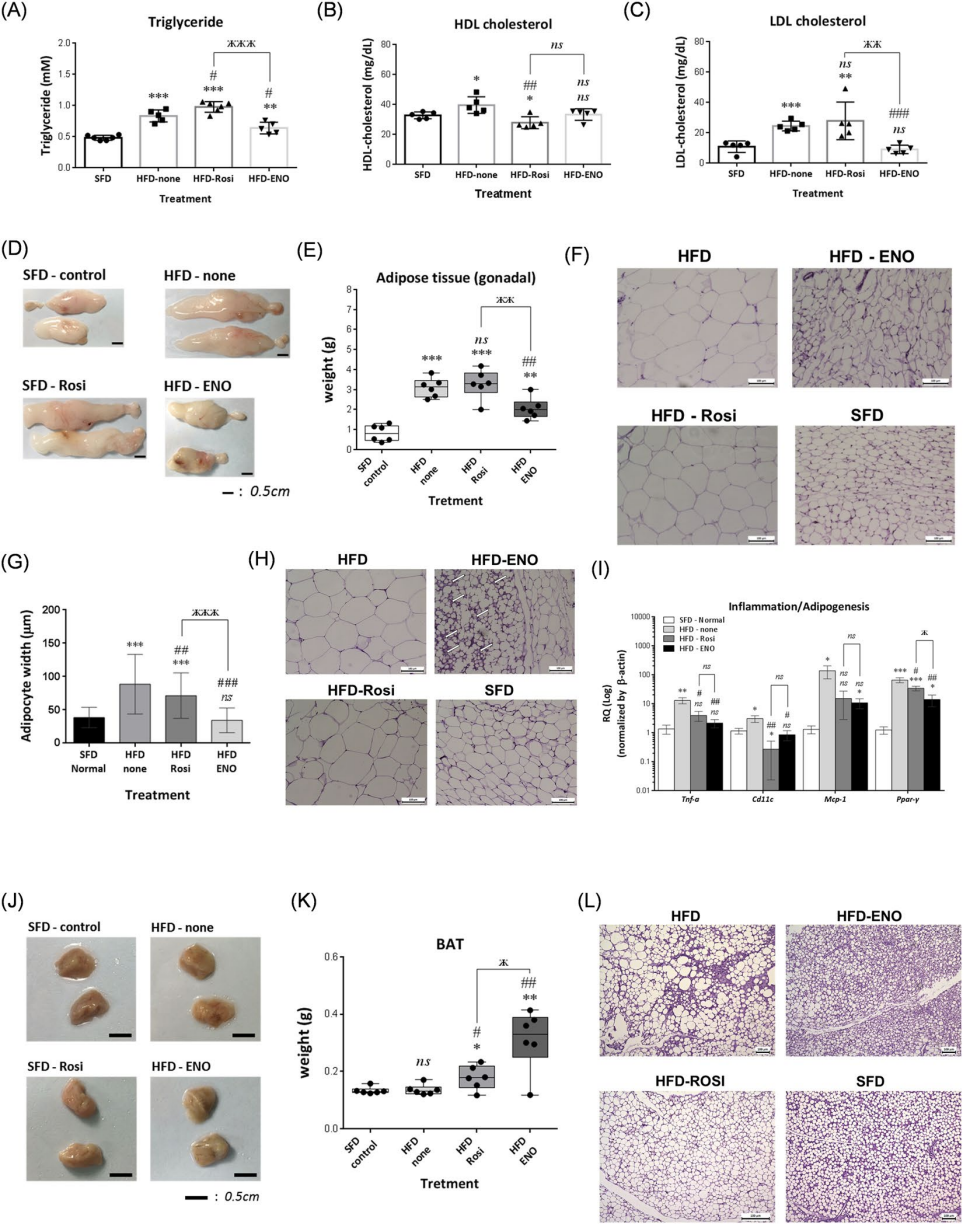
Effect of ENOblock treatment on adiposity in diet-induced obese mice. (**A**–**C**) Serum levels of triglyceride, HDL and LDL cholesterol in SFD, HFD, and HFD mice after 8 weeks’ treatment with ENOblock or rosiglitazone. n = 5–6. (**D**) Representative photographs of the dissected gonadal WAT. (**E**) Gonadal WAT weight. n = 6. (**F**) H&E stained gonadal adipose tissue to visualize adipocyte size distribution. (**G**) Variation in adipocyte width in the treated mice. n = 5 mice per treatment group were stained and 100 randomly selected adipocytes measured. (**H**) Indication of regions of beige-like adipocytes in H&E stained gonadal WAT from ENOblock treated HFD mice (indicated using white arrows). (**I**) qPCR analysis of the inflammatory markers *Tnf-α*, *Cd11c* and *Mcp-*1, and the master adipogenesis regulator, *Pparg* in gonadal WAT. Expression is compared using a log scale. n = 5. (**J**) Representative photographs of interscapular BAT. (**K**) Interscapular BAT weight. n = 6. (**L**) H&E stained interscapular BAT to visualize adipocyte size distribution. SFD = mice fed standard chow; HFD = high fat diet-fed mice; HFD-ENO = ENOblock treated HFD mice; HFD-Rosi = rosiglitazone treated HFD mice. For (**F**), (**H**) and (**L**); Scale bar = 100 µm. ns: not significantly different. *, ** or ***: significantly different from the corresponding ‘SFD-Normal’ or ‘SFD-Control’ (Standard Fat Diet-none-treated normal healthy mouse group) respectively with *p* < 0.05, *p* < 0.01 or *p* < 0.001; ## or ###: significantly different from the corresponding ‘HFD-none’ or ‘HFD-Control’ (HFD-non-treated control mouse group) sample with *p* < 0.01 or *p* < 0.001; ж, жж or жжж: significantly different from the corresponding ‘HFD-Rosi’ sample respectively with *p* < 0.05, *p* < 0.01 or *p* < 0.001.

HFD mice showed elevated serum levels of triglyceride, HDL cholesterol and LDL cholesterol after 8 weeks (Fig. 8A–C). HFD mice treated with rosiglitazone showed a further increase in serum triglyceride, reduced HDL cholesterol and no significant change in LDL cholesterol level. HFD mice treated with ENOblock showed reduced serum triglyceride and LDL cholesterol compared to untreated HFD mice. Serum LDL cholesterol in ENOblock-treated HFD mice reached the same range as SFD mice.

Representative photographs of gonadal WAT are shown in Fig. 8D. ENOblock treatment in HFD mice reduced gonadal tissue weight, whereas rosiglitazone treatment had no significant effect (Fig. 8E). Increases in adipocyte size in the gonadal WAT of HFD mice was prevented by ENOblock treatment, which was more effective than rosiglitazone at reducing adipocyte size (Fig. 8F,G). Modulators of the thermogenesis program in BAT and obesity have been shown to induce beige-like adipogenesis in WAT^33^. H&E staining revealed the infiltration of beige-like adipocytes in the gonadal WAT of ENOblock-treated HFD mice (Fig. 8I). ENOblock and rosiglitazone treatment also down-regulated expression of the inflammatory markers *TNF-α* and *Cd11c*, but not *Mcp-1* in gonadal WAT (Fig. 8I). Expression of the master mediator of adipogenesis*, Pparγ*^59^ was down-regulated in gonadal WAT by ENOblock treatment (Fig. 8I). Rosiglitazone treatment produced a smaller inhibition in *Pparγ* in HFD gonadal WAT compared to ENOblock.

Representative photographs of interscapular BAT are shown in Fig. 8J. HFD mice treated with ENOblock or rosiglitazone showed increased interscapular BAT weight compared to HFD or SFD mice (Fig. 8K). The effect of ENOblock on BAT weight was greater than that observed in the rosiglitazone-treated mice. H&E staining of interscapular BAT indicated reduced adipocyte size in ENOblock treated HFD mice compared to HFD, rosiglitazone-treated HFD and SFD mice (Fig. 8L). The markers of inflammation *Tnf-α*, *Cd11c* and *Mcp-1*, and the master regulator of adipogenesis *Pparγ* all showed down-regulated expression in HFD BAT after ENOblock or rosiglitazone treatment (Supplementary Fig. 6). Masson’s Trichrome staining demonstrated that ENOblock or rosiglitazone treatment inhibited the development of fibrosis in HFD BAT (Supplementary Fig. 7).

### ENOblock prevents the symptoms of pre-diabetes in obese mice at a comparable level to metformin

**Figure 9:**
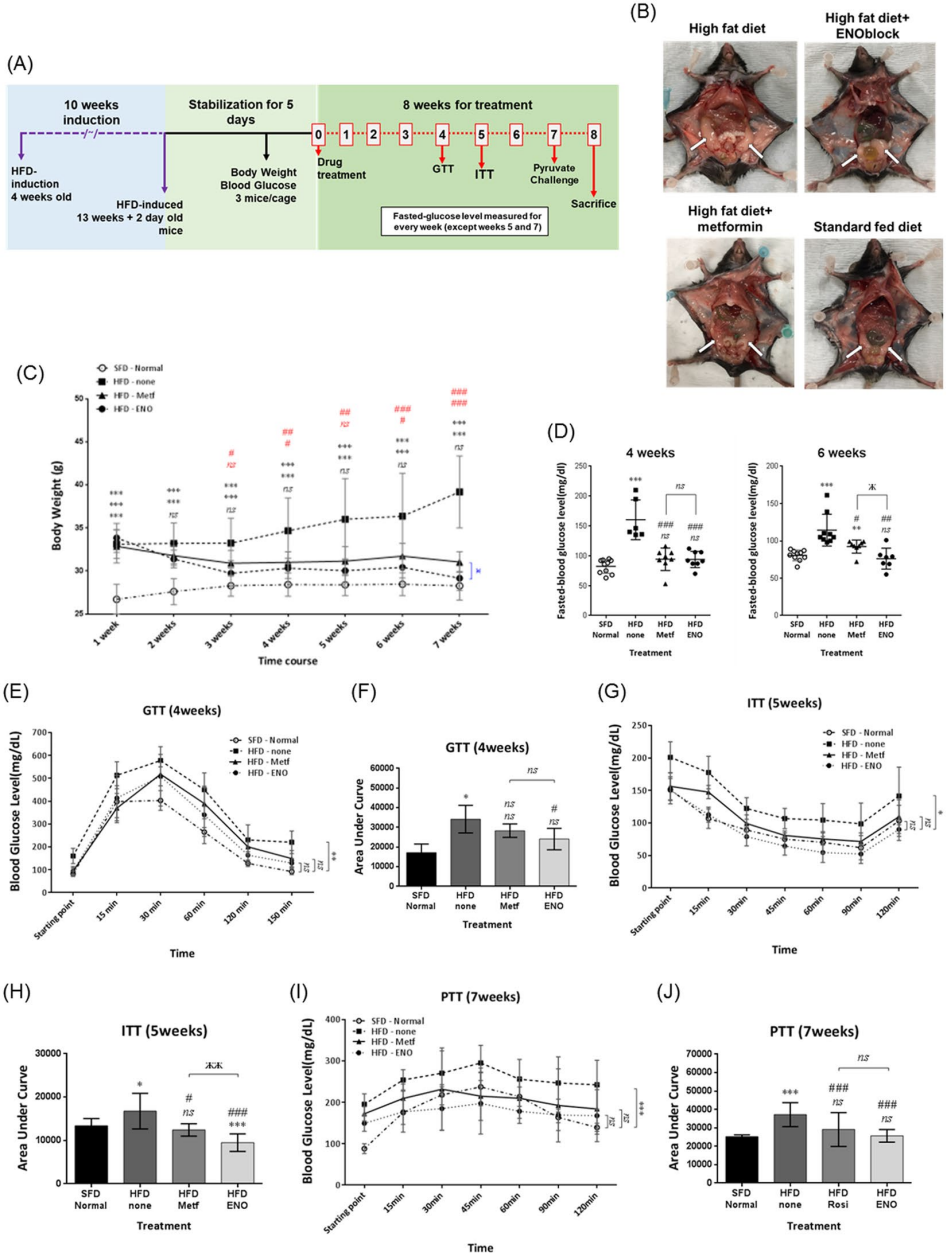
Comparison of ENOblock and metformin on parameters of obesity and pre-diabetes in diet-induced obese mice. (**A**) Schematic of the ENOblock and metformin treatment protocol in HFD mice. (**B**) Representative photographs of dissected mice to show visceral fat tissues (white arrows). (**C**) Body weight in the treated mice during drug treatment. (**D**) Fasted blood glucose at 4 and 6 weeks dug treatment. (**E**,**F**) GTT and area under the curve after 4 weeks drug treatment. (**G**,**H**) ITT and area under the curve after 5 weeks drug treatment. (**I**,**J**) PTT and area under the curve after 7 weeks drug treatment. SFD = mice fed standard chow; HFD = high fat diet-fed mice; HFD-ENO = ENOblock treated HFD mice; HFD-Metf = metformin treated HFD mice. n = 6–10; ns: not significantly different. *, ** or ***: significantly different from the corresponding ‘SFD-Normal’ or ‘SFD-Control’ (Standard Fat Diet-none-treated normal healthy mouse group) respectively with *p* < 0.05, *p* < 0.01 or *p* < 0.001; ## or ###: significantly different from the corresponding ‘HFD-none’ or ‘HFD-Control’ (HFD-none-treated control mouse group) sample with *p* < 0.01 or *p* < 0.001; ж, жж or жжж: significantly different from the corresponding ‘HFD-Metf’ sample respectively with *p* < 0.05, *p* < 0.01 or *p* < 0.001.

Obesity is associated with the development of prediabetes/insulin resistance, which also occurs in the diet-induced obesity mouse model^60^. To assess the potential effectiveness of ENOblock for preventing prediabetes in obesity, HFD mice were treated with ENOblock or metformin (Glucophage^TM^), which is a first line treatment for diabetes in obese patients^61,62^. The treatment schedule for ENOblock and metformin in HFD mice is shown in Fig. 9A. After 8 weeks, metformin and ENOblock treated HFD mice showed decreased quantities of visceral fat (Fig. 9B). During the course of drug treatment, both ENOblock and metformin reduced body weight. After 7 weeks, the ENOblock treated mice were significantly lighter than the mice treated with metformin (Fig. 9C). Fasted blood glucose was lowered in both metformin and ENOblock treated HFD mice, with ENOblock treated mice showing lower blood glucose levels than metformin treated mice at 6 weeks of drug treatment (Fig. 9D). A glucose tolerance test (at 4 weeks treatment), insulin tolerance test (at 5 weeks) and pyruvate tolerance test (at 7 weeks) indicated that ENOblock and metformin produce comparable improvements in insulin resistance/sensitivity and gluconeogenesis level, which all fell within the range observed in SFD mice (Fig. 9E–J).

### ENOblock treatment in obese mice reduced hyperinsulinemia and increased the population of anti-inflammatory M2 macrophages in the adipose tissue

**Figure 10:**
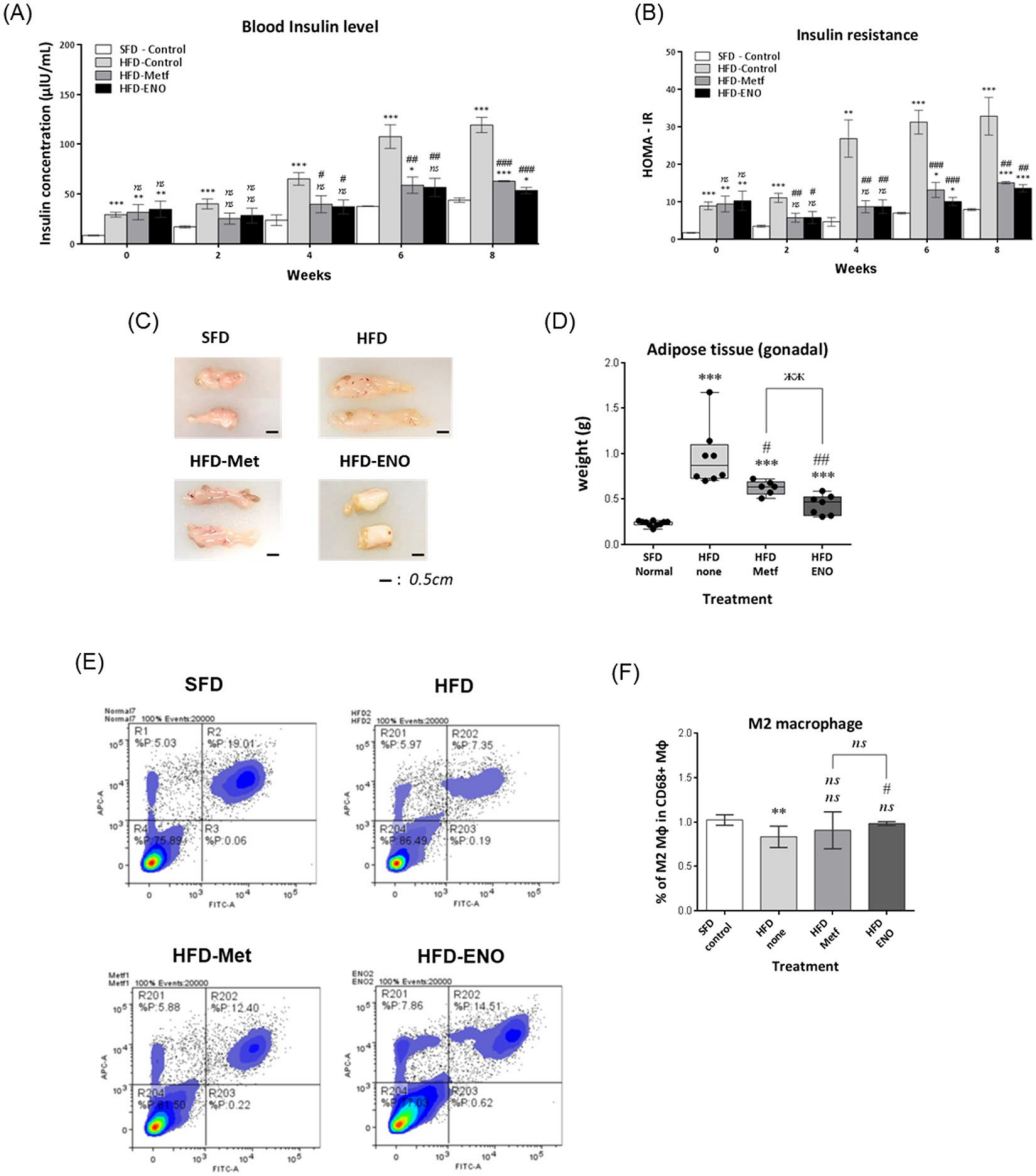
Effect of ENOblock or metformin treatment on HOM-IR and WAT inflammatory status in diet-induced obese mice. (**A**,**B**) Insulin serum level and insulin resistance in ENOblock or metformin treated HFD mice over 8 weeks of treatment. n = 6–10. (**C**) Representative photographs of dissected gonadal WAT. (**D**) Gonadal WAT weight in the treated mice. n = 6–10. (**E**) Representative flow cytometry plots of WAT-derived inflammatory cells stained with antibodies for the pan-macrophage marker, CD68 (macrosialin) and the M2 anti-inflammatory macrophage marker, CD206 (mannose receptor. (**F**) Quantification of the percentage of M2 type macrophages in the inflammatory population of WAT from the treated mice. n = 6. ns: not significantly different. *, ** or ***: significantly different from the corresponding ‘SFD-Normal’ or ‘SFD-Control’ (Standard Fat Diet-none-treated normal healthy mouse group) respectively with *p* < 0.05, *p* < 0.01 or *p* < 0.001; ## or ###: significantly different from the corresponding ‘HFD-none’ or ‘HFD-Control’ (HFD-none-treated control mouse group) sample with *p* < 0.01 or *p* < 0.001; ж, жж or жжж: significantly different from the corresponding ‘HFD-Metf’ sample respectively with *p* < 0.05, *p* < 0.01 or *p* < 0.001.

The HFD mice also displayed hyperinsulinemia and elevated HOMA-IR, which was decreased by ENOblock or metformin treatment (Fig. 10A,B). After 8 weeks’ treatment, ENOblock treatment produced a significantly greater reduction in hyperinsulinemia and HOMA-IR compared to metformin. Representative photographs of dissected gonadal WAT are shown in Fig. 10C. ENOblock and metformin treatment reduced gonadal WAT weight, with ENOblock producing a larger effect than metformin (Fig. 10D). Obesity is associated with adipose tissue inflammation, which is characterized by decreased numbers of anti-inflammatory ‘M2’ type macrophages^63,64^. Flow cytometry analysis of the expression of the pan-macrophage marker CD68 (macrosialin) and the M2 macrophage marker CD206 (mannose receptor) in WAT-derived inflammatory cells confirmed a reduction in M2 macrophage numbers in HFD mice compared to SFD mice. ENOblock treatment increased the proportion of M2 macrophages in the adipose tissue of HFD mice. Metformin treatment produced a variable response in the HFD mice and, overall, the proportion of M2 macrophages was not significantly changed compared to untreated HFD mice (Fig. 10E,F).

To further characterize the anti-obesity potential of ENOblock treatment, a short-term study was carried out in normal mice fed a HFD, to measure fecal fat content and cumulative food intake. The effect of ENOblock was compared with the clinically approved anti-obesity drug, orlistat^26^. It was observed that ENOblock or orlistat treatment reduced body weight gain (Supplementary Fig. 8A). Orlistat or ENOblock treatment also increased fecal fat content, although the effect of orlistat was much greater than ENOblock (Supplementary Fig. 8B). ENOblock, but not orlistat, treatment also reduced cumulative food intake in the treated mice after 20 days (Supplementary Fig. 8C).

## Discussion

The rising rates of obesity have become a major health concern. Developing novel therapeutics and new drug targets to treat obesity is a research priority. In this study, we investigated a novel small molecule enolase modulator, ENOblock, as a candidate drug and enolase-mediated transcriptional repression as a potential target for treating obesity and obesity-related complications.

Our study utilized the diet-induced model of obesity, in which mice are fed a highly palatable diet containing a high level of fats and sugar. Although this model is harder to establish than genetic models of obesity, it is considered superior for testing candidate therapeutics because it more closely resembles obesity in humans, which results from a combination of polygenic and environmental influences^60^.

The effect of ENOblock on adipogenesis was found to be dependent on the differentiation status of preadipocytes *in vitro* (Fig. 1). Key gene regulators of adipogenesis were found to be expressed in preadipocytes using qPCR. However, ENOblock treatment had a relatively minor effect on adipogenic gene expression in the preadipocytes (2 out 7 genes analyzed). In the presence of adipogenic factors that promote differentiation, ENOblock repressed all 7 adipogenic genes, irrespective of the treatment timing, i.e. before or after adding the adipogenic factors (Figs 1B and 2B). This contrasts with epigenetic factors that control adipogenesis, such as lysine-specific demethylase 1, which are only effective at the initiation of the adipogenic differentiation^33^. The ability of ENOblock to suppress adipogenesis was similar to the positive control compound, rapamycin, which also inhibited the expression of all 7 adipogenic genes (Fig. 2A,H,I). Moreover, our *in vitro* results indicate that ENOblock may promote mitochondrial membrane uncoupling and thermogenesis in preadipocytes via upregulation of *Ucp-1*, or *Ucp-3*, which was detected in preadiocytes and differentiating adipocytes, respectively (Fig. 1D,H). It has previously been reported that thermogenesis induction is also linked to the induction of regulators oxidative metabolism, such as *Nrf1*, *Cpt1b* and *Cox8b*^33^. Our results indicate the ENOblock treatment does not upregulate these three oxidative markers under non-adipogenic or adipogenic conditions (Figs 1C,G and 2C) or in brown preadipocytes (Supplementary Fig. 3C). However, the upregulation of uncoupling proteins by ENOblock appeared to be sufficient to induce mitochondrial inner membrane depolarization (Fig. 2E,F). Moreover, our positive control compound, forskolin, also induced membrane depolarization without upregulating *Nrf1*, *Cpt1b* and *Cox8b* (Fig. 2C). It should be noted that NaF, an inhibitor of enolase enzyme activity but not nuclear translocation, did not induce mitochondrial membrane depolarization or upregulate uncoupling proteins (Fig. 2D–F), implicating ENOblock-mediated targeting of enolase secondary functions as a mechanism producing depolarization.

In obese HFD mice, ENOblock reduced weight gain, and normalized both body weight and temperature to the range observed in SFD mice (Fig. 3C–E,G). However, it should be noted that dual-energy x-ray absorptiometry (DEXA) analysis would have been an ideal method for assessing fat re-distribution in ENOblock treated mice, and is essential for further assessment of the anti-obesity effects of this compound in animal models. These effects on body weight were achieved without influencing food intake, suggesting that the anti-obesity mechanisms of ENOblock as the inhibition of adipogenesis and enhancement of mitochondrial membrane depolarization in adipose tissues, rather than appetite suppression. ENOblock also suppressed the symptoms of pre-diabetes to a level comparable with the anti-diabetic drug, rosiglitazone (Figs 3H and 4A–H), although rosiglitazone did not produce anti-obesity effects in this model system.

In HFD mice liver, ENOblock reduced lipid accumulation, the development of steatosis and fibrosis, and the activation of stellate cells (Fig. 5D–I). In contrast, rosiglitazone did not reduce these pathological parameters. One potential explanation for these differences between the two drugs is the inability of rosiglitazone to down-regulate *Ap2* expression and the strong induction of *S100a9* expression observed in HFD mice (Fig. 6C,H). *Ap2* is a major inducer of adipogenesis and abundantly expressed in adipocytes, representing as much as 1–3% of the soluble protein, and *S100a9* regulates fibrosis because it stimulates myeloid inflammatory cells via toll-like receptor 4 and NF-κ-B^46,65^. Obesity is an inflammatory disease^66,67^ and ENOblock treatment reduced expression of the inflammatory markers *Il-6* and *Tnf-α* (Fig. 6C). Due to the mechanistic connection between obesity, inflammation and fibrosis, ENOblock can reduce fibrosis by targeting inflammatory responses in the HFD liver.

ENOblock treatment repressed the expression of *Srebp-1a* and *Srebp-1c*, which are major regulates of lipid homeostasis^40^, in the liver of HFD mice (Fig. 6D), providing a mechanistic explanation for the reduced adiposity observed in the ENOblock-treated mice. Reduced gene expression of *Srebp-1a* and *Srebp-1c* appears to be the main mechanism by which ENOblock represses these factors, because ENOblock treatment did not enhance the expression of *Amfr, Insig-1* and *Insig-2*, which would decrease the protein activity of *Srebp-1a* and *Srebp-1c* (Fig. 6E). This contrasts with known regulators of *Srebp*, such as betulin, a small molecule component of birch (Betula) tree bark, which block *Srebp* protein cleavage and activation^40^. ENOblock treatment disrupted *Srebp* expression without increasing expression of the LXR target genes, *Scap* and *Abcg5*, which may cause hepatic steatosis and hypertriglyceridemia when *Srebp* expression is inhibited by pharmacological ligands^40^ (Fig. 6F). However, it should be noted that while enolase siRNA treatment reduced enolase expression at both concentrations tested (40 and 60 pmol), *Srebp-1a*, *-1c*, and *-2* expression was only reduced at the 40 pmol concentration (Supplementary Fig. 2A,B). One possible explanation for this finding is that the different siRNA concentrations produced different effects on the cytoplasmic and nuclear distribution of enolase, and this should be addressed in subsequent studies of enolase-mediated regulation of *Srebp-1a*, *-1c*, and *-2* expression.

Obesity has been linked with impaired memory formation and hippocampal dysfunction^51^. All five markers of obesity-related inflammation in the hippocampus (*Il-6*, *Tnf-α*, *Cd11c*, *Tlr4* and *Nptx2*^51,52^) were down-regulated by ENOblock treatment (Fig. 6A). The expression of memory-associated genes in the hippocampus, such as *Creb* and *Tfam*, are tightly regulated to ensure correct establishment of long-term synaptic connections between neurons during memory formation^50,51^. In the hippocampus, *Creb* is an important sensor of energy status and functions in memory formation^56,68^. Obese mice in our study showed increased expression of hippocampal *Creb*, which has also been demonstrated previously^54^ and is known to interfere with memory formation^69^. ENOblock treatment reduced hippocampal *Creb* expression in obese mice (Fig. 7B). ENOblock treatment also produced a recovery in *Tfam* expression in HFD mice and increased hippocampal mitochondrial DNA content to the range observed in lean SFD mice (Fig. 7B,C). Although *Nrf-1* and *Nrf-2* expression showed opposite changes in obese mice (Fig. 7B), *Nrf-1* is thought to be the dominant factor determining *Tfam* promoter activity and mitochondrial biogenesis^58^. Although rosiglitazone treatment produced beneficial effects on inflammatory gene expression and mitochondrial DNA content, this drug appeared to produce neurotoxicity in the hippocampus, as indicated by a disrupted pattern of Nissl staining in the CA1 region (Fig. 7D). To our knowledge, this is the first demonstration that rosiglitazone can produce toxicity in the hippocampus. Overall, this data indicates that ENOblock treatment has the potential to correct the initiation of hippocampus impairment caused by diet-induced obesity. Further functional analyses, such as the Morris water maze, anxiety and anhedonia tests, would be needed to support this conclusion. We do not believe that the effects on gene expression in the brain are only restricted to the hippocampus. For example, the effect on ENOblock on suppressing the expression of inflammation-related genes was observed in the hippocampus, adipose and liver tissues. Therefore, other regions of the brain may show similar effects on gene expression.

The effect of ENOblock to down-regulate adipogenic gene expression *in vitro*, and *Srebp-1a* and *Srebp-1c* in liver tissue *in vivo*, was reflected in the marked reduction in the adiposity of ENOblock treated HFD mice (Fig. 8). Serum lipids, WAT mass and adipocyte size were significantly reduced (Fig. 8A–G). One goal in anti-obesity research is to develop drugs that mimic the normal cold exposure response by the central nervous system to produce ‘browning’ of white adipose tissue^70^. H&E staining of WAT indicated the presence of beige-like adipocytes in ENOblock treated mice, which was suggested by the mitochondrial depolarization observed in preadipocytes *in vitro* (Figs 2E,F and 8H). Compared to HFD mice, ENOblock treatment significantly upregulated *Ucp-3* in WAT and BAT (Supplementary Figs 5 and 6, respectively), which was also observed in differentiating preadipocytes *in vitro* (Fig. 2H). *Ucp-3* overexpression has been shown to produce fat-specific weight loss^71^. Therefore, we speculate that *Ucp-3* up-regulation in by ENOblock also contributes to reduced WAT mass and the appearance of beige-like adipocytes. In BAT, ENOblock treatment produced an increase in weight without increasing tissue size (Fig. 8J,K). This may be due to reduced lipid content and increased connective tissue in BAT after ENOblock treatment (Fig. 8L), which has been shown to increase BAT density^72^. Inflammatory regulators *Il-6* and *Tnf-α* were down-regulated by ENOblock in both WAT and BAT (Fig. 8I and Supplementary Fig. 6), providing a mechanistic explanation for the reduced BAT fibrosis in ENOblock treated mice (Supplementary Fig. 7).

We did not observe hepatotoxicity in the ENOblock treated group, as indicated by a significant reduction in alanine aminotransferase levels compared to high fat diet (HFD) mice, which fell to the range observed in SFD mice (Fig. 5C). In our previous study using a genetic model of type 2 diabetes (the db/db mouse), we assessed potential effects on genes linked to cardiotoxicity genes (*Kcnk1*, *Asah2*, *B4glant*, *MMP-3*), and reported no adverse effects after ENOblock treatment^7,73^. Although we observed no acute toxicity in the diet-induced obese mice after ENOblock treatment, we acknowledge that in-depth toxicological analysis should be an important component of future studies of ENOblock in animal models. For example, the weight loss produced by ENOblock treatment may be due to nausea or other non-specific effects, which would influence metabolism and decrease body weight.

In light of our finding that ENOblock improved both adiposity and the symptoms of obesity-related prediabetes, this drug was compared with metformin; the most commonly prescribed drug for this condition in patients^29–31^. Both drugs produced broadly similar effects on pre diabetic symptoms, although ITT (AUC), fasted blood glucose at 6 weeks and HOMA-IR were lower with ENOblock treatment (Figs 9D,F and 10A). Metformin treatment in patients has been observed to produce weight loss as a side effect^74^. In this diet-induced obesity model, ENOblock produced greater effects than metformin on whole body and WAT weight (Figs 9B,C and 10C,D). We also confirmed that anti-inflammation effect of this drug by demonstrating reduced inflammatory status in HFD WAT tissue (Fig. 10E,F). These results support the potential of ENOblock for development as an anti-obesity therapeutic.

It was previously shown that modulation of another glycolysis enzyme, glucokinase (hexokinase 4), by the compound PF-04991532 reduced hyperglycemia without causing hepatic steatosis in non-obese diabetic rats^75^. PF-04991532 enhances glucokinase activity and glycolysis, whereas ENOblock has been shown to not affect^76^ or reduce enolase enzyme activity^7,8,12^. This supports the modulation of non-enzymatic enolase transcriptional repression by ENOblock as the more likely mechanism for producing anti-obesity effects, because reduced enolase enzyme activity should inhibit glycolysis, which would decrease cellular glucose uptake and promote hyperglycemia. Moreover, PF-04991532 also increased plasma triglycerides, whereas ENOblock significantly reduced serum triglyceride and LDL cholesterol in our obesity model (Fig. 8A–C).

Enolase is a highly conserved, ancient enzyme that is widely expressed in all cells capable of glycolysis and fermentation. A number of glycolytic enzymes have been shown to moonlight in the cell nucleus and elucidation of the roles of these enzymes in the nucleus during pathological states has become a prominent area of research (reviewed in^9,77,78^). Recently, there is increased appreciation of the link between glycolytic enzyme nuclear translocation and metabolic status of cells, which regulates the cell response to environmental factors such as nutrient availability^9^. For example, pyruvate kinase translocates to the nucleus and induces the expression of glycolysis enzymes via binding to the master transcription factor, hypoxia-inducible factor-1α (HIF-1α)^9^. Gluconeogenesis can be considered the ‘reverse’ pathway of glycolysis. Therefore, the induction of glycolysis gene expression in cells would suppress gluconeogenesis. Our study and a recent report also shows that enolase nuclear translocation via ENOblock treatment also represses gluconeogenesis and down-regulates the master regulator, *Pck1* (Figs 4E–G and 6G). A greater understanding of the link between enolase nuclear translocation and peripheral oxidative capacity will be needed to fully interpret the utility of this mechanism for treating metabolic disorders and obesity.

**Figure 11:**
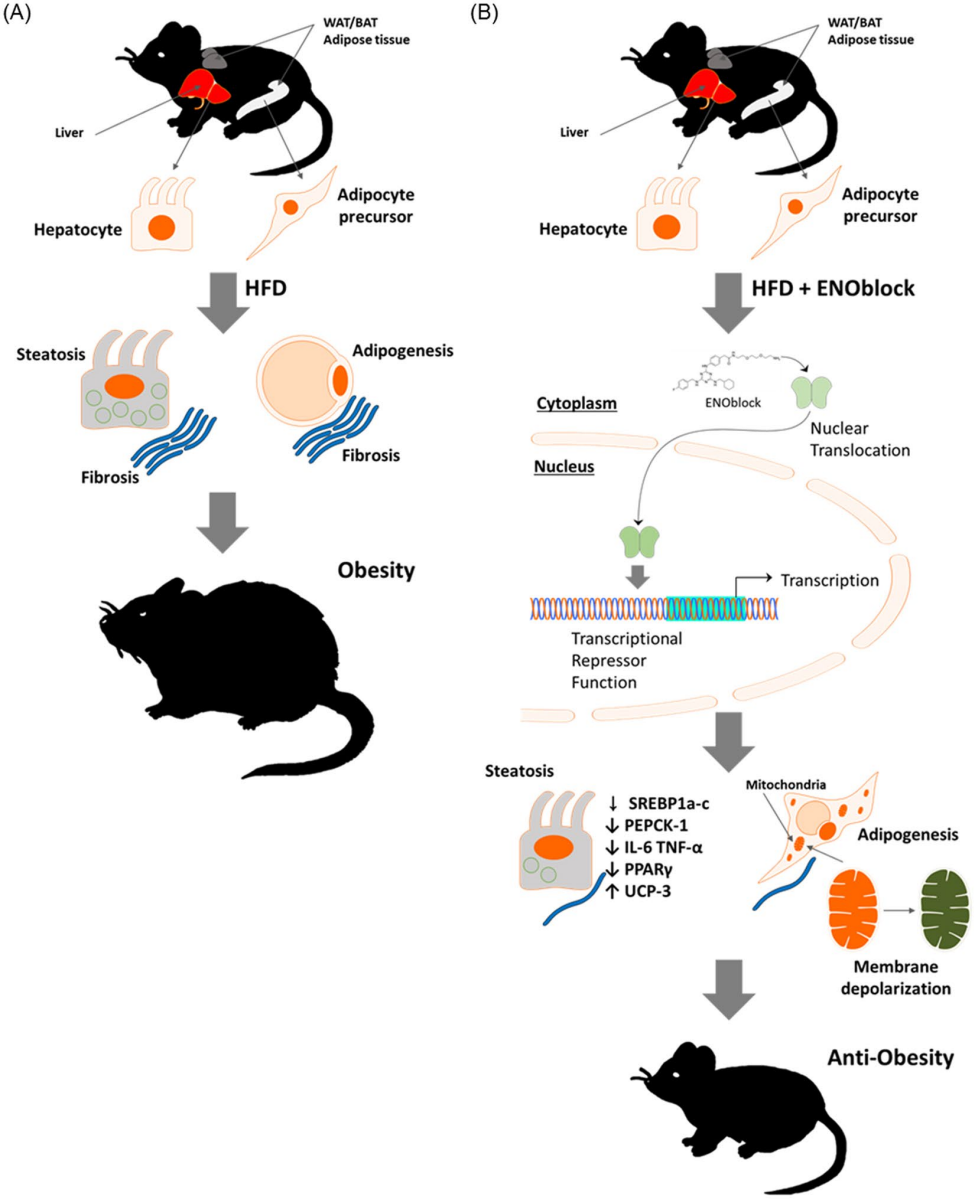
Schematic of the consequences of ENOblock treatment on the progression of diet-induced obesity. In HFD mice, ENOblock treatment induces the nuclear localization of enolase and its secondary moonlighting activity as a transcriptional repressor. Key regulators of adiposity, gluconeogenesis and inflammatory response are downregulated, with a concomitant increase in adipose tissue mitochondrial membrane depolarization via *Ucp-3* upregulation. This combined pattern of gene expression modulation inhibits the progression of obesity and related complications. The copyright holder (Mrs Hyunju Park) has granted permission to Springer Nature Limited to publish the images of the mice in Fig. 11 of the manuscript entitled “ENOblock inhibits the pathology of diet-induced obesity” by Cho, *et al*., under a CC BY open access license.

In summary, obesity is a disease that has become a major global medical and economic burden. Current pharmacological approaches to treat obesity have only achieved limited success and produced side effects^79^. In this study, the compound ENOblock, a modulator of enolase moonlighting as a transcriptional repressor, ameliorated hyperglycemia and reduced adiposity in a diet-induced model of weight gain that closely resembles the pathogenesis of human obesity. ENOblock treatment inhibited gluconeogenesis, adiposity and obesity-related inflammation via concomitant repression of the regulatory genes *Pck-1*, *Srebp-1a* and *Srebp-1c*, and *Tnf-a* and *Il-6*. These genetic alterations also produced mitochondrial uncoupling and the recovery of down-regulated *Ucp-3* in WAT. A schematic of the major findings in this study is presented in Fig. 11. Overall, these results suggest ENOblock as a candidate therapeutic and enolase sub-cellular localization (moonlighting) as a target for anti-obesity drug development.

## Materials and Methods

### Reagents and Antibodies

ENOblock(N-[2-[2-(2-aminoethoxy)ethoxy]ethyl]-4-[[4-[(cyclohexylmethyl)amino]-6-[[(4-fluorophenyl)methyl]amino]-1,3,5-triazin-2-yl]amino]-benzeneacetamide hydrochloride) was designed and synthesized by Professor Young-Tae Chang, Pohang University of Science and Technology, Republic of Korea and synthesized Professor Jin Hee Ahn, Gwangju Institute of Science and Technology, Republic of Korea, in accordance with the published protocol^6^. Dexamethasone, forskolin, rapamycin and rosiglitazone were purchased from Santa Cruz Biotechnology (CA, USA). Metformin (1,1-dimethylbiguanide hydrochloride), orlistat, OSMI-1, 3-isobutyl-1-methylxanthine (IBMX), sodium fluoride (NaF), oil red O and an antibody for α–smooth muscle actin (catalogue number A5228) were purchased from Sigma-Aldrich (MO, USA). Tetramethylrhodamine, ethyl ester, perchlorate (TMRE) was purchased from Thermo Fisher Scientific (MA, USA).

### Cell Culture

3T3-L1 murine preadipocytes were obtained from the Korean Cell Line Bank (Seoul National University, Republic of Korea) and cultured in DMEM supplemented with 10% calf serum, 50 units mL^−1^ penicillin and 50 µg mL^−1^ streptomycin (PenStrep). 3T3-L1 cells were induced to differentiate into adipocytes follows: 48 h post-confluent cells (designated day 0) were cultured in DMEM supplemented 10% FBS, 0.5 mM IBMX, 2 µg/ml dexamethasone, 1 µg/mL insulin and PenStrep for 2 days. Thereafter, the cells were incubated with fresh DMEM supplemented with 10% FBS and 1 µg/mL insulin. Hep G2 human hepatocytes were obtained from the Korean Cell Line Bank and cultured in DMEM supplemented 10% FBS and 1% PenStrep.

### Isolation of primary preadipocytes

8 week old male C57BL/6 J mice were purchased from Damool Science, Republic of Korea. The protocol to isolate primary preadipocytes was based on a previously published methodology^33^. In brief, mice were sacrificed and entire white gonadal tissue or interscapular brown adipose tissue were dissected and homogenized in 1 mL DPBS containing 0.5% BSA. Tissues were then treated with 0.8 U/mL collagenase and 2.7 U/mL dispase in 3 mL digestion media, with incubation at 37 °C for 30 min. The volume was adjusted to 10 mL with digestion media and a final concentration of 10 mM EDTA. The suspension was passed through a 70 µm filter into new 50 mL tube. The lipid portion was removed by centrifugation at 500 *g* for 10 min at 4 °C. Preadipocytes in the stromal vascular fraction of the pellet were isolated by treatment with RBC lysis buffer on ice for 5 min. Lysis was stopped with MACs buffer (PBS with 2% FBS and 1 mM EDTA). Preadipocytes were washed once more with PBS solution, counted and seeded on a culture plate. To induce differentiation, the preadipocytes were treated with the adipogenic factors, 0.5 mM IBMX, 2 µg/mL dexamethasone, 10 µM rosiglitazone and 1 µg/mL insulin, as previously described^33^.

### RNA extraction from cells and tissues

RNA was extracted with the TRIzol reagent (Thermo Fisher Scientific, MA, USA), in accordance with the manufacturer’s instructions. Prior to RNA extraction, tissues were frozen in liquid nitrogen and ground into powder with a glass mortar.

### Quantitative real-time PCR

For quantitative real-time PCR, the transcript level of genes of interest was measured using a StepOnePlus Real Time PCR System (Applied Biosystems, UK). Total RNA was reverse transcribed into cDNA using an AccuPower® RT PreMix (Bioneer Corporation). The cDNA was used for real-time PCR according to the manufacturer’s guidelines, with the following modifications: PCR was performed in triplicate in a total volume of 20 μL 2X Power SYBR® Green PCR Master Mix(Applied Biosystems, UK) containing 200 nM final concentration of the specific primer and 1 μL cDNA. The mixture was incubated for 10 min at 95 °C prior to the PCR amplification, which consisted of 40 cycles of denaturation, annealing and extension. Denaturation was performed for 15 s at 95 °C, annealing was performed for 1 m at 60 °C and extension was performed at 72 °C for 20 s with fluorescence detection at 72 °C after each cycle. After the final cycle, the melting-point analysis of the samples was performed within the range of 60–95 °C with continuous fluorescence detection. A specific cDNA sample was included in each run and used as a reference for comparison between runs. The expression level of 18 s rRNA or actin or was used for normalization while calculating the expression levels of the other genes (as indicated in the text). Results were presented as the relative expression level of each gene. The primers used in this study are shown in Supplementary Table 1. To measure the relative amount of mRNA expression, the Δ CT value of each gene is calculated with respect to the CT values of β-actin or 18S rRNA (internal control) in each sample. Next, the Δ CT value of each gene in the drug treated samples were further normalized with the Δ CT value of the same gene in the non-treated HFD sample or SFD sample to calculate the ΔΔ CT value, indicating the relative expression of each gene in the drug treated samples compared with that of the controls. The relative mRNA expression fold-change was finally calculated using the 2^−2ΔΔCT^ method. The final mRNA expression in each experiment is calculated as the average value of three independent set of experiments.

### Western blotting

Protein was harvested from 10–30 mg tissue samples using 1 ml cold homogenization buffer (20 mM Tris-Cl (pH 7.4), 0.32 M sucrose and 1 mM EGTA containing protease inhibitor cocktail) and homogenization with a Wheaton® Potter-Elvehjem Tissue Grinder (NJ, USA). Protein was quantified using the Bradford assay. 30 to 40 μg protein samples were loaded into 10% polyacrylamide gels, and transferred to nitrocellulose membranes after electrophoresis. Primary antibody was used at a 1:1000 dilution in TBS-T plus 5% skimmed milk, and incubated with the membranes overnight at 4 °C. Primary antibody detection was performed with a HRP-conjugated secondary (anti-mouse IgG-HRP, sc2031, Santa Cruz Biotech, CA, USA) used at a 1: 10000 dilution, with incubation for 30 min at room temperature. Expression signals were visualized with an ECL solution (RPN2232, GE Healthcare Life Science, UK). The bands were visualized using the ImageQuant LAS 500 (GE Healthcare Life Sciences).

### Measurement of mitochondrial membrane potential

Mitochondrial membrane potential in live cells was assessed using the TMRE probe, as previously described^41^. Cells were seeded in 96 well plates and treated with compound of interest for 72 h. Cells were then treated with 1 µM TMRE for 20 min at 37 °C. The cells were then washed with PBS containing 0.2% bovine serum albumin (BSA) and fluorescence was measured with a microplate reader (λ_ex_ = 549 nm, λ_em_ = 575 nm; SpectraMax, Molecular Devices, CA, USA). For live cell imaging, cells were seeded in 96-well clear bottom black polystyrene microplates (Corning™, catalog no. 07-200-565) at a density of 5 × 10^3^ cells and treated with compound of interest for 72 h. Cells were then treated with 200 nM TMRE for 20 min at 37 °C, washed with 0.2% BSA in PBS and imaged and quantified TMRE stained cells with a Lionheart FX automated microscope (BioTek, VT, USA). Fluorescence was measured with an excitation of 549 nm and an emission of 575 nm (Texas-Red filter). In order to account for variations in cell location in the well the fluorescence was measured with a 2 × 2 area scan and the results averaged. The mean objective intensity was measured and normalized by cell counting in each well using Gen5TM 3.0 software (BioTek, VT, USA). Data is represented as the mean of 9 determinations.

### Oil Red O staining

Differentiating 3T3-L1 adipocytes in 12 well culture dishes were stained with Oil Red O to visualize lipid accumulation using the previously published protocol^80^. Oil Red O staining was quantified by dissolving the cells in 1 mL 100% isopropanol for 5 min at room temperature with gentle rocking. 200 µL aliquots were transferred to a 96 well plate and absorbance was measured at λ = 492 nm (SpectraMax, Molecular Devices, CA, USA).

### Immunocytochemistry analysis of enolase nuclear translocation

Hepatocytes were cultured in MultiCell^TM^ 8B, staining chambers (CtrlBio). The enolase antibody H-300 (Santa Cruz Biotechnology sc-15343; 1:50 dilution) was used for immunocytochemistry. Goat Anti-Rabbit IgG-heavy and light chain Antibody DyLight®488 Conjugated (Bethy) was the secondary antibody. Stained cells were mounted with ProLong™ Gold Antifade Mountant with DAPI. Images were detected using a Fluoview FV 1000 confocal laser-scanning microscopy with 40x magnification. Two randomly selected high-power fields were quantified from each sample of stained cells and averaged to obtain the value for each slide; n = 3 for each experimental group and analyzed by ImageJ. Nuclear enolase in the cells and their ‘Integrated Density’ was used for calculating the corrected total cell fluorescence (CTCF = Integrated Density - (Area of selected cell X Mean fluorescence of background readings)).

### siRNA silencing of enolase expression

siRNA-mediated knockdown of enolase expression was assessed using qPCR. Hep G2 hepatocytes were seeded in 6 well plates at a cell density of 2 × 10^5^ cells per well. Twenty-four hours after seeding, cells were transfected with 40 or 60 pmol siRNA, following the manufacturer’s protocol provided by Santa Cruz Biotechnology. Transfected cells were used for SREBP and enolase gene expression analysis at twenty-four hours’ post-transfection.

### Chromatin Immunoprecipitation Assay

A chromatin immunoprecipitation (ChIP) assay kit was purchased from Cell Signaling (CST - SimpleChIP® Enzymatic Chromatin IP Kit) and the assay was carried out following the manufacturer’s instructions. The enolase antibody H-300 was used for the assay (Santa Cruz Biotechnology). Precipitated DNAs were analyzed using the following primers: SREBP1 Primers: Transcription start site (TSS) including primer ACCTGTGCCCACTTCTTTGC and GCCAGGTGCCCAGTAAATGA; TSS non-including primer (down-stream) AGTGACGGCTAGGGCTCCTT and CTCTACCCATGGCGGTTCCT; TSS non-including primer (up-stream) CCCTCACCCCACCATTAGC and GCCAATGGAGTTTTGAAATCG. SREBP2 primers: TSS including primer TGAGTTTGTGATGCTCTTATGCATT and TTGGGTTGGCTTTCTTTTGG; TSS non-including primer (down-stream) GATCTTGGCTCACTGCAACCT and GATGTAGTGTTGCGTGCCTGTAA; TSS non-including primer (up-stream) CCCAAGAGACAATAAAAATCCATCA and GCATAAGAGCATCACAAACTCATGA.

### Animal studies

The studies were carried out in accordance with the Institute for Laboratory Animal Research Guide for the Care and Use of Laboratory Animals and were approved by the Gwangju Institute of Science and Technology Animal Care and Use Committee (approval number: GIST-2017-079). High fat diet-fed male C57BL/6 J mice were purchased from Charles River, Japan. The mice had been fed the HFD from 4 weeks old and were supplied at 14 weeks old. After delivery, the mice continued the HFD (supplied by Jung Ahn Laboratory Animal, Inc., Republic of Korea). The animals were stabilized in the animal facility for 5 days and maintained in a 12 h/12 h light cycle at a density of 3 mice per cage. The mice had free access to the HFD chow, which was weighed beforehand. The cages were cleaned weekly before the fasting experiment. After stabilization, the drug treatment regime was initiated.

For the first experiment, the mice were divided into three groups of 6 mice and treated with drug for 8 weeks, as follows: Group (1) 8 mg/kg rosiglitazone; Group (2) 12 mg/kg ENOblock; Group (3) vehicle alone (saline with 10% DMSO). It should be noted that the micromolar dose for rosiglitazone is higher than ENOblock: 22.4 mM and 20.2 mM, respectively. Drug was administered every 24 h via intraperitoneal injection with a solution volume of 10 uL/g. Food intake and body weight was monitored weekly from week 1 of drug treatment. Fasted blood glucose was measured at weeks 4, 6 and 8. Blood glucose was measured with a OneTouch Ultra (LifeScan, CA, USA). The insulin tolerance test (ITT), glucose tolerance test (GTT) and pyruvate tolerance test (PTT) were carried out using the guidelines provided by the Mouse Metabolic Phenotyping Centers, Yale School of Medicine (MMPC; https://www.mmpc.org/). GTT, ITT and PTT was carried out after 4, 5 and 7 weeks of drug treatment, respectively.

For the second experiment, the HFD mice were divided into three groups, as follows: Group (1) 8 mice received 120 mg/kg metformin; Group (2) 7 mice received 12 mg/kg ENOblock; Group (3) 8 mice received vehicle alone (saline with 10% DMSO). The selected metformin dose was based on a previously published study^81^. Drug was administered every 24 h for 8 weeks, via intraperitoneal injection with a solution volume of 10 uL/g. Food intake and body weight was monitored weekly from week 1 of the drug treatment. GTT, ITT and PTT were carried out after 4, 5 and 7 weeks of drug treatment, respectively. For the animal experiments, blinding was used when carrying out the GTT, ITT and PTT.

At the end of drug treatment, the mice were sacrificed by inhalation of diethyl ether. Blood was collected from the heart, and the kidneys, liver, brain, spleen, pancreas, skeletal muscle, gonadal adipose tissue and brown adipose tissue were harvested. The blood was placed in a microfuge tube and left at 15 min at room temperature to undergo clotting. The clot was removed using centrifugation (1500 *g* at 4 °C for 10 min). The supernatant was divided into 50 μL aliquots and frozen at −80 °C. The dissected organs and tissues were washed twice with PBS and stored at −80 °C.

As a short-term test to compare ENOblock and orlistat in mice fed a HFD, male C57BL/6 J mice were divided into three groups of 5 mice, stabilized in the animal facility for 7 days, and fed a HFD for 20 days while receiving the following drug regimes: (1) 10 mg/kg ENOblock by daily IP delivery; (2) 15 mg/kg orlistat by daily oral gavage; (3) Untreated. During the drug treatment and feeding with a HFD, the mice were assessed for body weight (at days 0, 4, 8 and 12), cumulative food intake (at days 4, 8, 12, 16 and 20) and fecal fat content (at days 4, 8 and 12, which was measured using a previously published protocol^82^).

### Measurement of serum triglyceride

Blood samples were collected from mice and centrifuged using serum separation tube (BD Microtainer® SSTTM, NJ, USA). The serum samples were stored at −80 °C before tested. Triglyceride quantification was determined with a colorimetric Triglyceride Quantification Kit (K622- 100; BioVision, CA, USA) in accordance with the manufacturer’s instructions. Triglyceride concentration was calculated and expressed as mM. Blood serum samples were used from 5 to 6 animals per treatment group in duplicate.

### Measurement of serum HDL and LDL cholesterol

High-density lipoprotein (HDL) and low-density lipoprotein (LDL) cholesterols were measured with a HDL and LDL/VLDL Quantification Colorimetric/Fluorometric Kit (catalog # K613, BioVision, Inc., USA), using the colorimetric assay. Blood serum samples were used from 5 animals per treatment group in duplicate.

### Serum insulin quantification

Levels of insulin in the sera was measured using a mouse ELISA kit (Abnova, Taiwan). The serum was diluted 10-fold for the ELISA. Blood serum samples were used from 6 animals per treatment group.

### Measurement of serum alanine aminotransferase (ALT) activity

ALT activity was expressed as nmol/mon/mL (=mU/mL) and the assay was carried out following the method provided by the kit (catalog #K752, BioVision, Inc., USA). Blood serum samples were used from 6 animals per treatment group in triplicate.

### Embedding and sectioning of tissues

Tissues from the dissected mice were washed 2 times with PBS, blotted dry, placed into a cryo-mold and covered with OCT for embedding (Leica, Germany). Embedded tissues were then snap-frozen using liquid nitrogen and transferred to slurry of isopropanol. The frozen tissues were stored at −80 °C until sectioning using CM 1850 cryostat (Leica, Germany).

For paraffin sections, white and brown adipose tissues were formalin-fixed with 10% formaldehyde solution for 5 hr, and processed through paraffin embedment after dehydration and xylene-washing processes. Paraffin embedded blocks were sectioned at 3 μm. Sections were deparaffinized before H&E or Masson’s trichrome staining. Hematoxylin and eosin staining was carried out using a staining kit (Merck, Germany).

5 mice per treatment group were used to analyze liver fibrosis, lipid accumulation, steatosis and hepatic satellite numbers. Microscopic images were captured at x200 magnification.

### Measurement of lipid accumulation in liver tissue

Liver lipid accumulation was visualized using oil red O staining and measured with ImageJ 1.45 s software (NIH, USA). Liver tissue was sectioned at 8 μm thickness, air dried for 10 minutes and fixed in 10% formalin solution. Slides were rinsed with tap water, washed with 60% isopropanol, stained with freshly prepared oil red O staining solution (3 g/L) (Sigma-Aldrich, USA) for 15 min, rinsed with 60% isopropanol and mounted with aqueous mountant (Sigma-Aldrich, USA). 30 randomly selected lipid droplets in 5 randomly captured microscope images from 5 mouse livers/treatment group were used for the quantification (magnification: 200x).

### Measurement of liver steatosis

Steatosis was visualized using H&E staining, as previously described^83^. 10 different regions of each mouse liver (magnification 200x) were used to quantify hepatic steatosis with the Image J software 1.48 v software (NIH, USA),

### Determination of tissue fibrosis

Tissue sections were stained with Masson Trichrome Staining Kit (Sigma-Aldrich, USA). Fibrotic regions were quantified by detecting the blue stained area with the Image J software (NIH, USA), as described previously^84^. 8 to 10 randomly captured microscope images from sections, prepared from 5 mouse livers (cryo-sectioned) or BAT (paraffin-sectioned) per treatment group were used for the quantification (magnification: 200x).

### Detection of hepatic stellate cells

Hepatic stellate cells were detected by α-SMA staining, as previously described^45^. Stellate cells were quantified by counting α-SMA and DAPI double-labeled cells. Ten randomly captured microscope images of cryo-sections from 5 mouse livers in each treatment group was used for the quantification (magnification: 200x).

### Quantification of hippocampus mitochondrial DNA

Mitochondrial DNA (mtDNA) content in the hippocampus was quantified using a previously published methodology^54^. Phenol-chloroform-isoamyl alcohol was used to purify DNA and 2 ng was loaded into each well of a 384-well plate with TaqMan primers for 18S nuclear (Mm03928990_g1) or 16S mitochondrial (Mm04260181_s1) DNA (Applied Biosystems, USA). Hippocampus mtDNA was quantified from 6 mice in each treatment group.

### Nissl staining of hippocampal neurons

Neurons in the hippocampus were visualized using a NovaUltra Nissl staining kit (IHC World, USA). 4 sections of the hippocampus from 6 SFD, 5 HFD, 6 rosiglitazone-treated and 5 ENOblock-treated HFD mice were used for staining.

### Measurement of adipocyte size

Adipose tissue sections were stained with H&E and the size distribution of adipocytes was measured using Image J software, using a previously described method^85^. Gonadal adipose tissue from 5 mice per treatment group was stained and 100 randomly selected adipocytes were measured from each mouse.

### Isolation of adipose tissue immune cells for flow cytometry

The isolation of immune cells from adipose tissue was based on the previously published protocol^86^. Gonadal adipose tissue was dissected from the sacrificed mice. After being weighed and photographed, the adipose tissue was digested in collagenase. Gonadal tissue from 6 mice per treatment group were used for immune cell analysis. The stromal vascular fraction pellet containing immune cells was suspended in FACS buffer (1X DPBS (without calcium and magnesium), 2 mM EDTA, and 1% FCS) and stained with antibodies against CD68 (macrosialin) and CD206 (mannose receptor). The populations of stained cells were analyzed with a FACS CantoII (BD Biosciences, USA) and Flowlogic software (Miltenyi Biotech, Republic of Korea).

### Statistical analysis

The Student’s t test (Microsoft Excel 2013), 1-way-ANOVA with Dunnett’s multiple comparison test or 2-way-ANOVA with Tukey’s multiple comparison test (Figs 3E–G, 4A–C,G, 9C,E,G,I and 10A,B) as the post-test analysis (Graphad Prism version 6) was used for comparison between experimental groups, as indicated in the manuscript figure legends. *p*-values of <0.05 were considered significant. Unless otherwise stated, all results are the average of three independent experiments and the error bars are standard deviation.

## Electronic supplementary material

Supplementary information
