# Supplementary material for: ENOblock inhibits the pathology of diet-induced obesity
Source: Sci Rep. 2019 Jan 24;9:493. doi: 10.1038/s41598-018-36715-3 (PMC6346001; doi:10.1038/s41598-018-36715-3)
Supplement: Supplementary file 1 — Supplementary information [file 41598_2018_36715_MOESM1_ESM.pdf]

ENOblock inhibits the pathology of diet-induced obesity

Haaglim Cho[1], Ji-Hyung Lee[1], JungIn Um[1], Sunwook Kim[1], Yukyung Kim[1], Woong-Hee Kim[1], Yong Sook Kim[2], Haushabhau S. Pagire[3], Jinhee Ahn[3], Youngkeun Ahn[2], Young-Tae Chang[4,5], Da-Woon Jung[1]*, and Darren R. Williams[1]*

[1]New Drug Targets Laboratory, School of Life Sciences, Gwangju Institute of Science and Technology, 1 Oryong-Dong, Buk-Gu, Gwangju 61005, Republic of Korea

[2]Cell Regeneration Research Center, Department of Cardiology, Cardiovascular Center, Chonnam National University Hospital, 671 Jebong-ro, Dong-gu, Gwangju, 501-757, Republic of Korea.

[3]Department of Chemistry, Gwangju Institute of Science and Technology, 1 Oryong-Dong, Buk-Gu, Gwangju 61005, Republic of Korea

[4]Center for Self-assembly and Complexity, Institute for Basic Science (IBS), Pohang 37673, Republic of Korea

[5]Department of Chemistry, Pohang University of Science and Technology (POSTECH), Pohang 37673, Republic of Korea

*To whom correspondence should be addressed: 1) Da-Woon Jung, School of Life Sciences, Gwangju Institute of Science and Technology, 1 Oryong-Dong, Buk-Gu, Gwangju 61005, Republic of Korea. Tel: +82-62-715-3554; email: jung@gist.ac.kr 2) Darren R. Williams, School of Life Sciences, Gwangju Institute of Science and Technology, 1 Oryong-Dong, Buk-Gu, Gwangju 61005, Republic of Korea. Tel: +82-62-715-2509; email: darren@gist.ac.kr

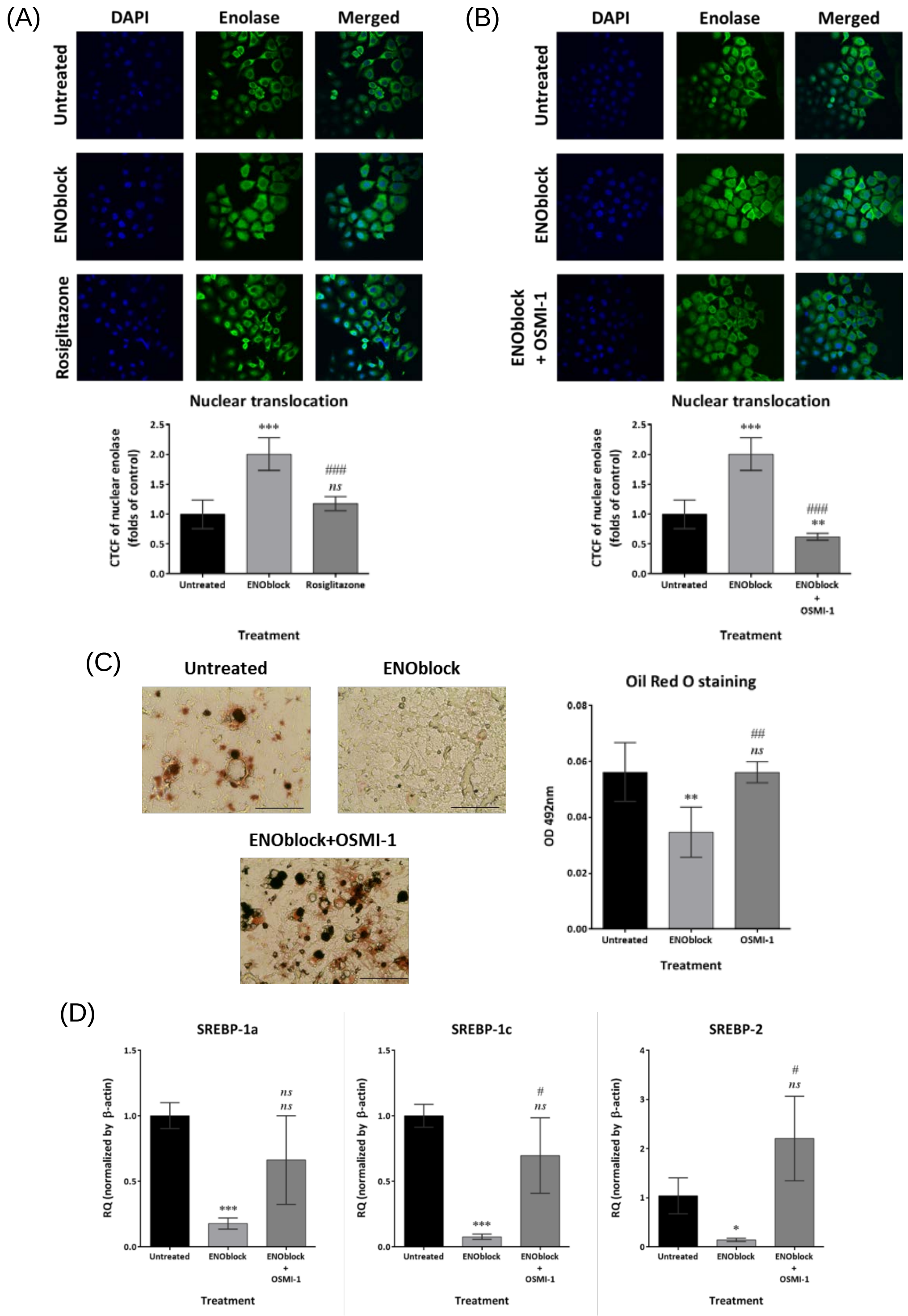

Supplementary Figure 1. (A) Enolase immunostaining of Hep G2 hepatocytes treated with 10 µM ENOblock or 10 µM rosiglitazone for 24 h. Cells were counter-stained with DAPI to visualize cell nuclei. The bar chart shows quantification of enolase staining in the hepatocyte nuclei. (B) Enolase immunostaining of Hep G2 hepatocytes treated with 10 µM ENOblock or 50 µM OSMI-1 (an inhibitor of *O*-GlcNAc modification, which is linked to enolase nuclear translocation) for 24 h. The bar chart shows quantification of enolase staining in the hepatocyte nuclei. n=3. ns: not significantly different. ** or ***: significantly different from the corresponding 'Control.' respectively with $p< 0.01$ or $p<0.001$; ###: significantly different from the ENOblock treated group with $p<0.001$. (C) Microscopic images of oil red O staining in 3T3-L1 preadipocytes treated with 10 µM ENOblock and/or 50 µM OSMI-1 for 72 h and adipogenic factors for 5 days. Scale bar=100 µm. The bar chart shows oil red O staining quantification. ns: not significantly different; **: significantly different from the corresponding 'Untreated' with $p< 0.01$; ##: significantly different from the corresponding 'ENOblock' sample with $p<0.01$. (D) qPCR analysis of SREBP-1a, -1c and SREBP-2 expression in Hep G2 hepatocytes treated with with 10 µM ENOblock and/or 50 µM OSMI-1 for 72 h. n=9; ns: not significantly different; *** or *: significantly different from the corresponding 'Untreated' with $p< 0.001$ or $p<0.05$; #: significantly different from the corresponding 'ENOblock' sample with $p<0.05$.

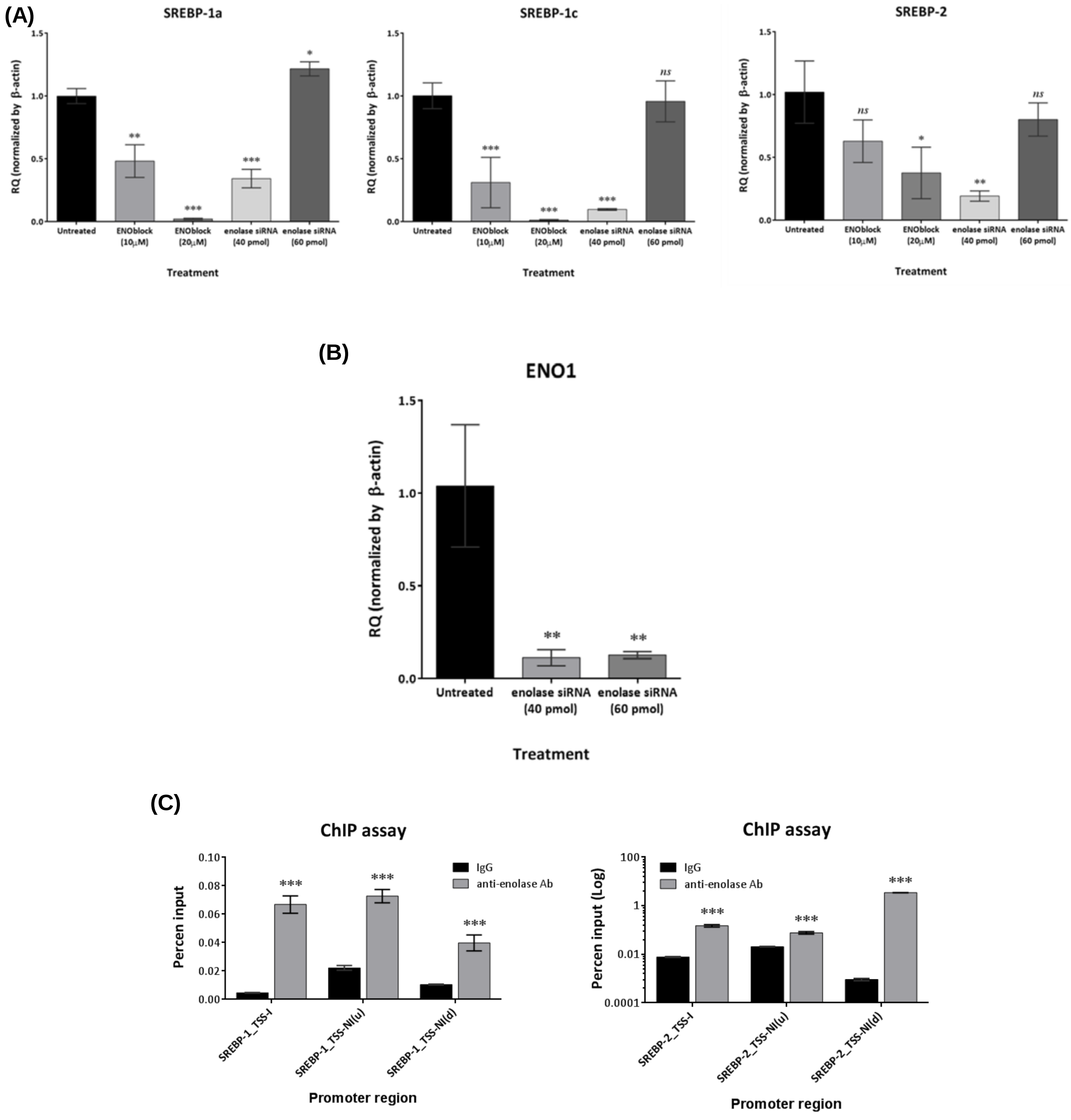

Supplementary Figure 2. (A) qPCR analysis of SREBP-1a, -1c and SREBP-2 expression in Hep G2 hepatocytes treated with 40 or 60 pmol enolase siRNA for 24 h. (B) qPCR analysis of enolase expression in Hep G2 hepatocytes treated with 40 or 60 pmol enolase siRNA for 24 h. (C) ChIP analysis of enolase binding to the SREBP-1 and -2 upstream promoter in Hep G2 cells treated with 10 μM ENOblock for 24 h. PCR product signals obtained from the SREBP-1 and -2 samples are shown. ***: significantly different from the corresponding 'IgG' with $p < 0.001$.

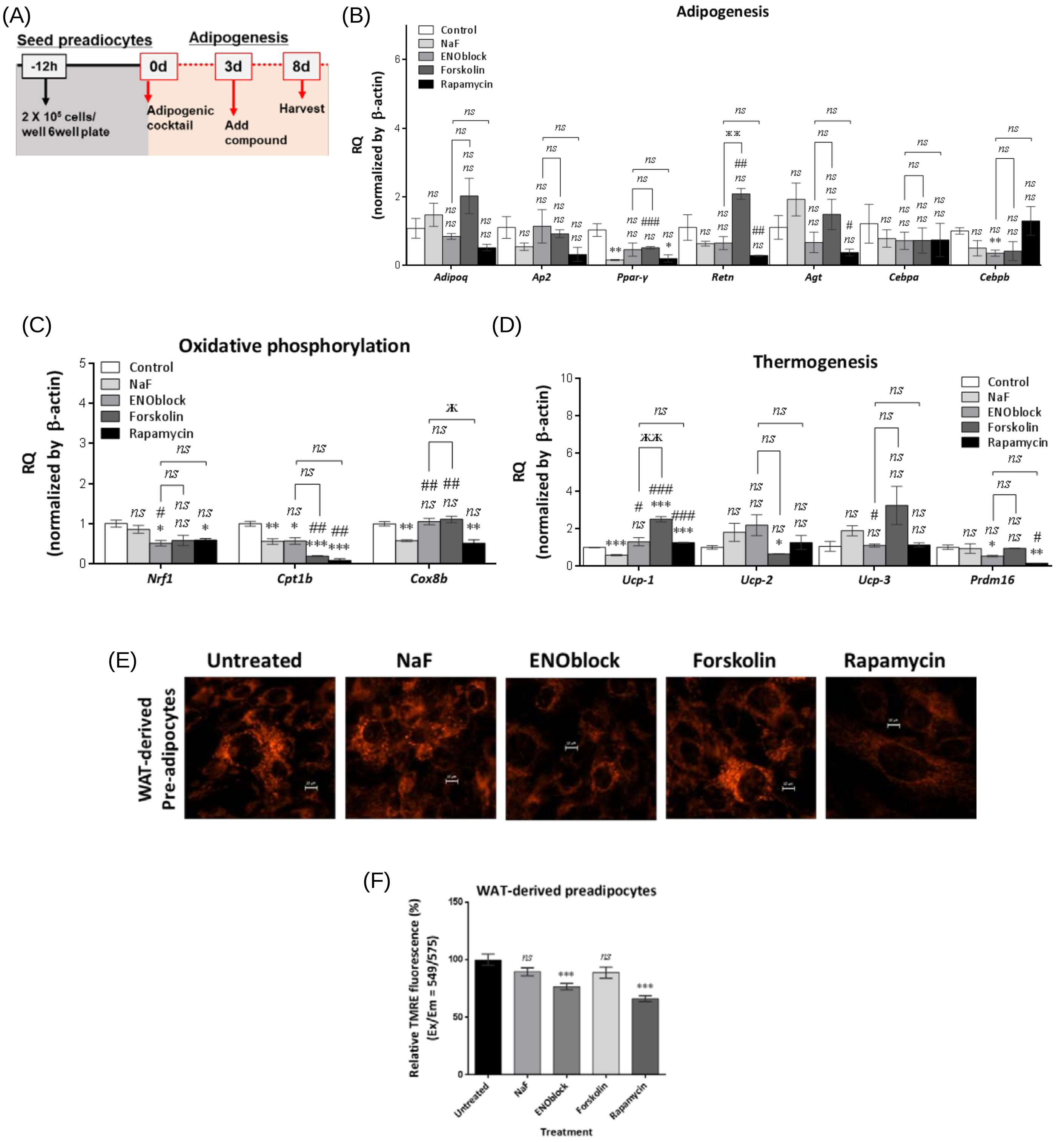

Supplementary Figure 3. (A) Schematic of the compound treatment protocol in primary cultures of differentiating brown adipocytes. (B) Effect of 72 h treatment with 10 µM forsoklin, 1 µM rapamycin,10 µM ENOblock or 1 mM NaF on the expression of adipogenesis regulatory genes in differentiating adipocytes. (C) Expression of oxidative phosphorylation regulatory genes. (D) Expression of thermogenesis regulatory genes. (E) Live cell imaging of TMRE fluorescence to visualize mitochondrial membrane potential in primary WAT preadipocytes after treatment with 10 µM ENOblock, 1 mM NaF, 10 µM forsoklin or 1 µM rapamycin for 72 h. (F) Quantification of mitochondrial membrane potential in WAT preadipocytes. For *in vitro* analysis: For (B-D) n=3. ns: not significantly different. *, ** or ***: significantly different from the corresponding 'Control' or 'Untreated' respectively with $p<0.05$, $p<0.01$ or $p<0.001$; ## or ###: significantly different from the corresponding 'NaF' sample with $p<0.01$ or $p<0.001$; ж, жж or жжж: significantly different from the corresponding 'ENOblock' sample respectively with $p<0.05$, $p<0.01$ or $p<0.001$.

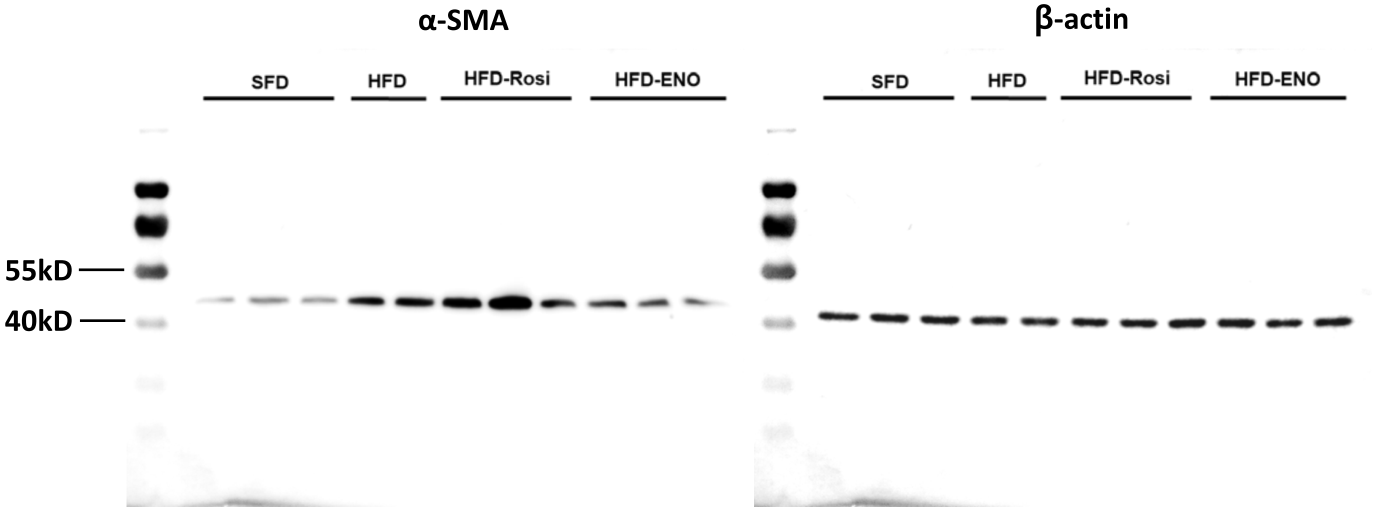

Supplementary Figure 4. Western blot analysis of α-SMA (45 kD) expression in liver tissue from HFD mice treated with ENOblock or rosiglitazone. SFD=mice fed standard chow; HFD=high fat diet-fed mice; HFD-ENO=ENOblock treated HFD mice; HFD-Rosi=rosiglitazone treated HFD mice. β-actin (42 kD) expression was used as a loading control. The blot was visualized on ImageQuant LAS 500 (GE Healthcare Life Sciences).

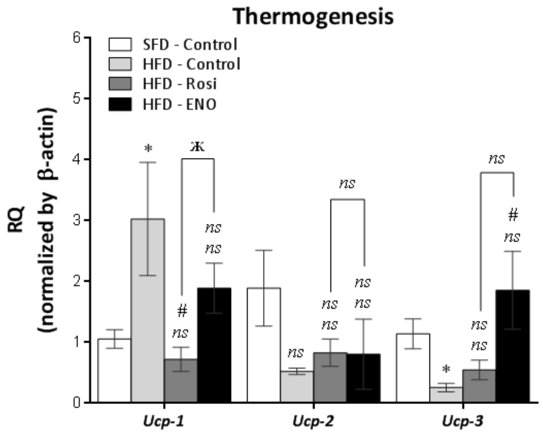

Supplementary Figure 5. qPCR analysis of the thermogenesis related genes *Ucp-1*, *Ucp-2* and *Ucp-3* expression in gonadal WAT of HFD mice after treatment with ENOblock or rosiglitazone. SFD=mice fed standard chow; HFD=high fat diet-fed mice; HFD-ENO=ENOblock treated HFD mice; HFD-Rosi=rosiglitazone treated HFD mice. n=3. ns: not significantly different. *, ** or ***: significantly different from the corresponding 'SFD-Normal' or 'SFD-Control' (Standard Fat Diet-none-treated normal healthy mouse group) respectively with $p<0.05$, $p<0.01$ or $p<0.001$; ## or ###: significantly different from the corresponding 'HFD-none' or 'HFD-Control' (HFD-none-treated control mouse group) sample with $p<0.01$ or $p<0.001$; ж, жж or жжж: significantly different from the corresponding 'HFD-Rosi' sample respectively with $p<0.05$, $p<0.01$ or $p<0.001$.

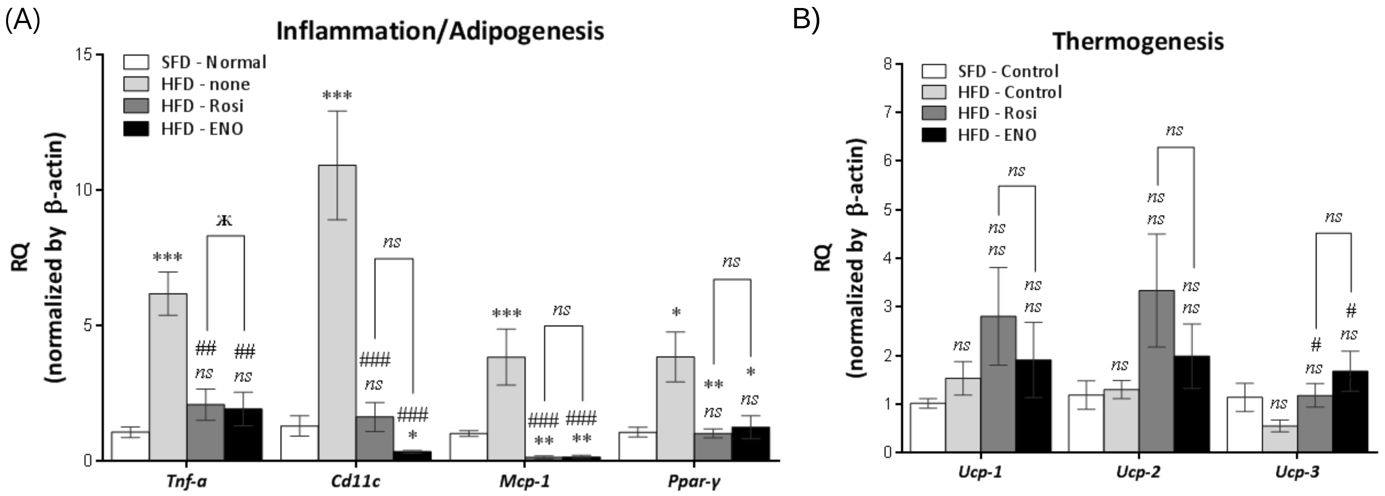

Supplementary Figure 6. (A) qPCR analysis of the inflammatory markers *Tnf-α*, *Cd11c* and *Mcp*-1, and the master adipogenesis regulator, *Pparg* in interscapular BAT of HFD mice after treatment with ENOblock or rosiglitazone. (B) Expression of the thermogenesis related genes *Ucp-1*, *Ucp-2* and *Ucp-3* in BAT. SFD=mice fed standard chow; HFD=high fat diet-fed mice; HFD-ENO=ENOblock treated HFD mice; HFD-Rosi=rosiglitazone treated HFD mice. n=3. ns: not significantly different. *, ** or ***: significantly different from the corresponding 'SFD-Normal' or 'SFD-Control' (Standard Fat Diet-none-treated normal healthy mouse group) respectively with $p<0.05$, $p<0.01$ or $p<0.001$; ## or ###: significantly different from the corresponding 'HFD-none' or 'HFD-Control' (HFD-none-treated control mouse group) sample with $p<0.01$ or $p<0.001$; ж, жж or жжж: significantly different from the corresponding 'HFD-Rosi' sample respectively with $p<0.05$, $p<0.01$ or $p<0.001$.

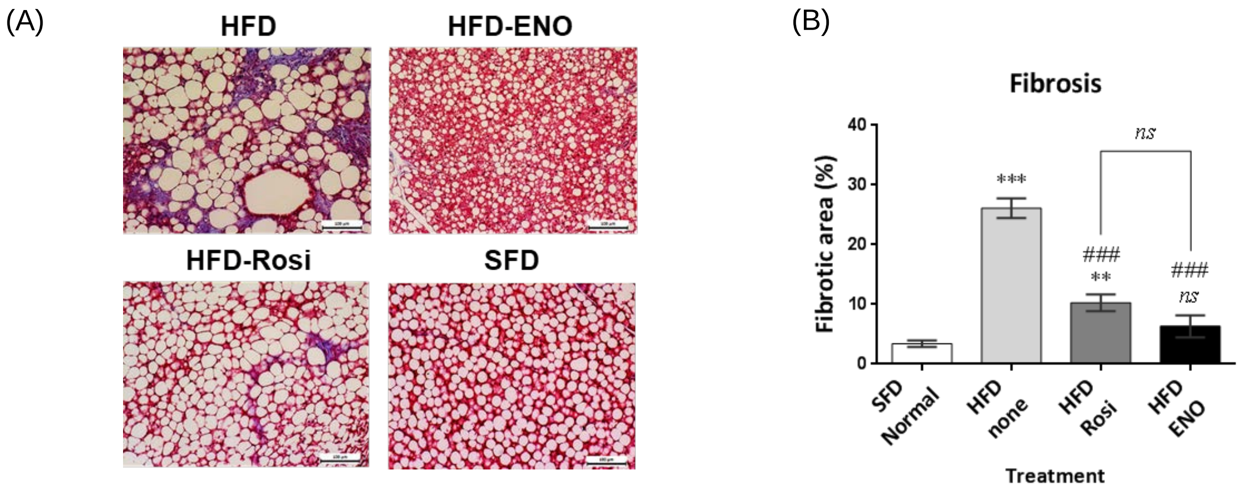

Supplementary Figure 7. (A) Representative images of Masson's Trichrome staining to indicate fibrosis (blue color) in interscapular BAT tissue. Scale bar=100 µm. (B) Quantification of fibrosis in BAT. SFD=mice fed standard chow; HFD=high fat diet-fed mice; HFD-ENO=ENOblock treated HFD mice; HFD-Rosi=rosiglitazone treated HFD mice. n=8 to 10 randomly captured microscope images from sections, prepared from 5 mouse BAT/treatment group. ns: not significantly different. *, ** or ***: significantly different from the corresponding 'SFD-Normal' or 'SFD-Control' (Standard Fat Diet-none-treated normal healthy mouse group) respectively with $p<0.05$, $p< 0.01$ or $p<0.001$; ## or ###: significantly different from the corresponding 'HFD-none' or 'HFD-Control' (HFD-none-treated control mouse group) sample with $p<0.01$ or $p<0.001$; ж, жж or жжж: significantly different from the corresponding 'HFD-Rosi' sample respectively with $p< 0.05$, $p<0.01$ or $p<0.001$.

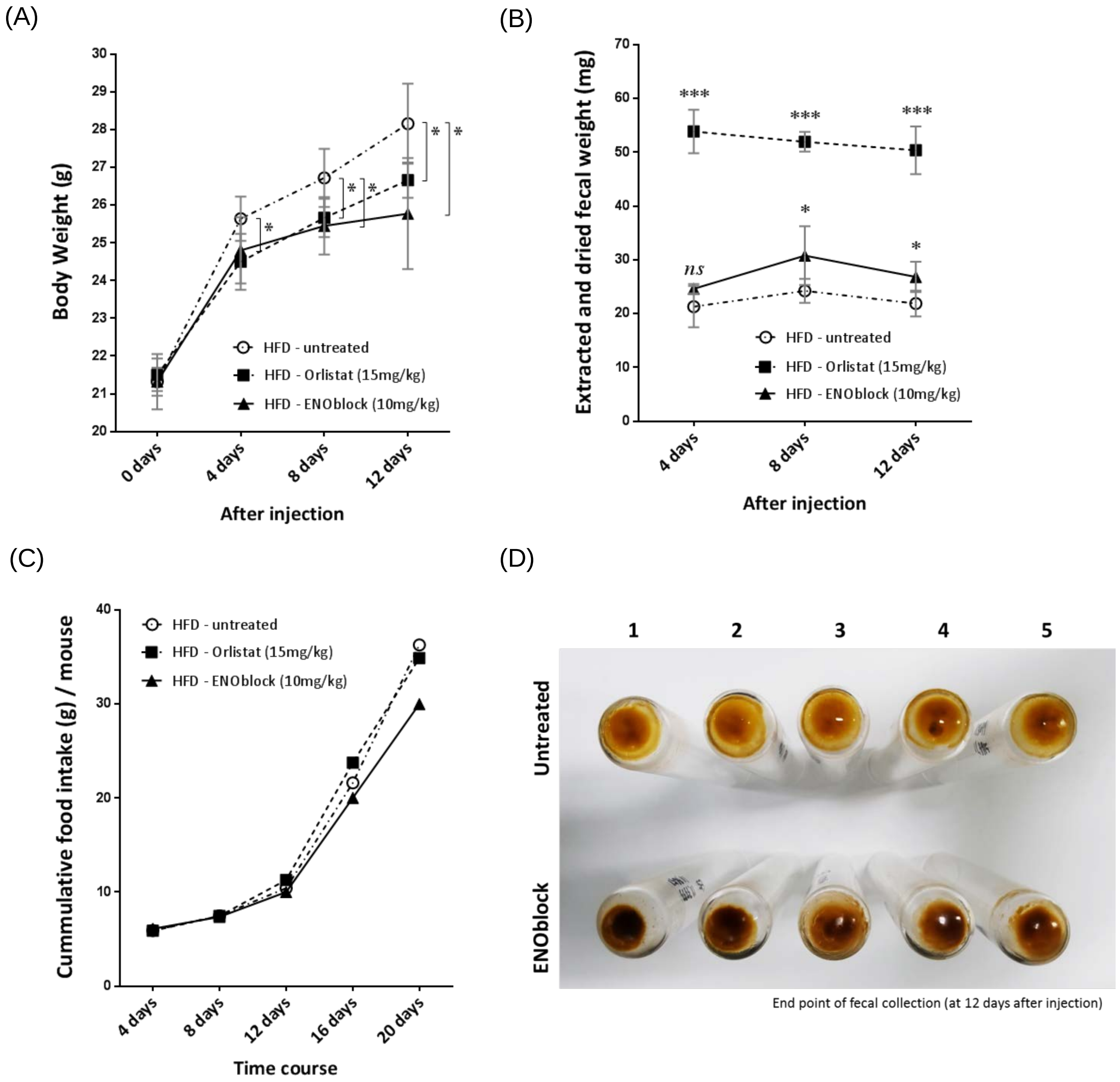

Supplementary Figure 8. (A) Body weight in C57Bl6 mice exposed to a HFD and treated with 8 mg/kg ENOblock IP or 15 mg/kg orlistat by oral gavage. (B) Fecal lipid content in the treated mice. (C) Cumulative food intake in the treated mice. (D) Photograph of the fecal lipid preparation from untreated and ENOblock treated mice at 12 days of drug treatment. n=5; ns: not significantly different; *** or *: significantly different from the corresponding ‘Untreated’ with $p< 0.001$ or $p<0.05$.

Supplementary Table 1: Primer sequences used in this study

Gene	Strand	Primer Sequences	RefSeq
Abcg5	F	CGTGGCGGACCAAATGA	NM_031884
	R	GCTCGCCACTGGAAATTCC	
Adipoq	F	AACCCCTGGCAGGAAAGG	NM_009605
	R	TGAACGCTGAGCGATACACAT	
Agt	F	AGTGGGAGAGGTTCTCAATAGCA	NM_007428
	R	GACGTGGTCGGCTGTTCCT	
Amfr	F	GGCAGGCCTCGCTTGAA	NM_011787
	R	ATCCGGGACCCATCGAA	
Ap2	F	CCGCAGACGACAGGAAGGT	NM_024406
	R	AGGGCCCCGCCATCT	
a-SMA	F	TCCCTGGAGAAGAGCTACGAACT	NM_007392
	R	AAGCGTTCGTTTCCAATGGT	
Cd11c	F	CTGGATAGCCTTTCTTCTGCTG	NM_021334
	R	GCACACTGTGTCCGAACTCA	
Cebpa	F	GAGCTGAGTGAGGCTCTCATTCT	NM_001287514
	R	TGGGAGGCAGACGAAAAAAC	
Cebpb	F	CGCAACACACGTGTAACTGTCA	NM_009883
	R	AACAACCCCGCAGGAACAT	
Cox8b	F	GGTTCCCAAAGCCCATGTC	NM_007751
	R	CGGCGGAAGTGGGAGTTT	
Cpt1b	F	GTGCAAGCAGCCCGTCTAG	NM_009948
	R	TTGCGGCGATACATGATCAT	
Il-6	F	TAGTCCTTCCTACCCCAATTTCC	NM_000600
	R	TTGGTCCTTAGCCACTCCTTC	
Insig-1	F	CACCTGGGAGAACCACACAAG	NM_153526
	R	CACGGCAATACAGCGCATAA	
Insig-2	F	ATGTGATCACGAGCATCTTTTCA	NM_001271531
	R	GGCTGTGCCGCAGCAT	
Mcp-1(=Ccl2)	F	CTCCTGCTCATAGCTACCACCAT	NM_011331
	R	GGGTGCTCACCGCATCTG	
Nptx2	F	GCCACGCAGGCCTTTG	NM_016789
	R	GAGGACGCGGTCCCATATG	
Nrf-1	F	CAGACACGTTTGCTTCGGAAA	NM_001164226
	R	CCCACTCGCGTCGTGTACT	
Nrf-2	F	CGTGAGTCCTGGTCATCAAAAA	NM_010902
	R	GAGCCTCTAAGCGGCTTGAA	
Pck-1	F	CTGCATAACGGTCTGGACTTC	NM_011044
	R	CAGCAACTGCCCGTACTCC	
Pck-2	F	CCACAGGACTCCCCATGCT	NM_028994
	R	ATGGCTGCTATGTACCTCCC	

Pgc-1a	**F**	CCGTAGGCCCAGGTACGA	NM_008904
	**R**	TGCGGTATTCATCCCTCTTGA	
Pgc-1β	**F**	TCGAAATCTCTCCAGTGACATGA	NM_133249
	**R**	CTACAATCTCACCGAACACCTCAA	
Ppar-γ	**F**	GCCCACCAACTTCGGAATC	NM_001127330
	**R**	TGCGAGTGGTCTTCCATCAC	
Prdm16	**F**	GAGAGCTGCATCAAAAAGCAGAT	NM_027504
	**R**	CGCCTGATTGGCATCCA	
Retn	**F**	TTCCTGATGTCGGTCAGTTGAG	NM_022984
	**R**	TCCCCGTCCCTGTCAACA	
S100a9	**F**	TCATGGAGGACCTGGACACA	NM_001281852
	**R**	CAGCATCATACACTCCTCAAAGCT	
Scap	**F**	TCTGACTTCTTCCTCCAGATGCT	NM_001001144
	**R**	CATCCGGCGAATGTCGAT	
Srebp-1a	**F**	GATGTGCGAACTGGACACAG	NM_011480
	**R**	CATAGGGGGCGTCAAACAG	
Srebp-1c	**F**	CCAGAGGGTGAGCCTGACAA	NM_001313979
	**R**	AGCCTCTGCAATTTCCAGATCT	
Tfam	**F**	GCACCCTGCAGAGTGTTCAA	NM_009360
	**R**	CGCCCAGGCCTCTACCTT	
Tlr-4	**F**	CCTGGCTGGTTTACACGTC	NM_138557
	**R**	GACATTGCAGAAACATTCGC	
Tnf-α	**F**	AAGCCTGTAGCCCACGTCGTA	NM_001278601
	**R**	GGCACCACTAGTTGGTTGTCTTTG	
Ucp-1	**F**	CACCTTCCCGCTGGACACT	NM_009463
	**R**	CCTGGCCTTCACCTTGGAT	
Ucp-2	**F**	CCTCAAAGCAGCCTCCAGAA	NM_011671
	**R**	CAATCGGCAAGACGAGACAGA	
Ucp-3	**F**	CCACCTTAGGGCAAGAACGA	NM_009464
	**R**	AGATGAGAAAACCTCCGAGAGAGA	
